# A List of Candidate Cancer Biomarkers for Targeted Proteomics

**Published:** 2007-02-07

**Authors:** Malu Polanski, N. Leigh Anderson

**Affiliations:** The Plasma Proteome Institute, P.O. Box: 53450, Washington DC, 20009-3450, USA

**Keywords:** cancer, biomarkers, targeted proteomics, validation

## Abstract

We have compiled from literature and other sources a list of 1261 proteins believed to be differentially expressed in human cancer. These proteins, only some of which have been detected in plasma to date, represent a population of candidate plasma biomarkers that could be useful in early cancer detection and monitoring given sufficiently sensitive specific assays. We have begun to prioritize these markers for future validation by frequency of literature citations, both total and as a function of time. The candidates include proteins involved in oncogenesis, angiogenesis, development, differentiation, proliferation, apoptosis, hematopoiesis, immune and hormonal responses, cell signaling, nucleotide function, hydrolysis, cellular homing, cell cycle and structure, the acute phase response and hormonal control. Many have been detected in studies of tissue or nuclear components; nevertheless we hypothesize that most if not all should be present in plasma at some level. Of the 1261 candidates only 9 have been approved as “tumor associated antigens” by the FDA. We propose that systematic collection and large-scale validation of candidate biomarkers would fill the gap currently existing between basic research and clinical use of advanced diagnostics.

## Introduction

The study of cancer biomarker proteins began in 1847 with the discovery by Henry Bence-Jones of what turned out, more than 100 years later, to be a tumor-produced free antibody light chain “Bence Jones protein” in the urine of a multiple myeloma patient (Bence-Jones 1847; Kyle 1994) where it was present in large quantities and could be revealed by simple heat denaturation. One hundred and 40 years later this protein was demonstrated to be present also in the serum (Sinclair et al. 1986), and in 1998 a routine immunodiagnostic test was approved by the FDA. Hormones produced by tumors were also detected early on (Chan and Sell 1999): adrenocorticotropic hormone (ACTH), calcitonin, and chorionic gonadotropin (hCG), for example, are elevated in specific cancer types, though not with the tumor specificity of Bence-Jones proteins.

Unfortunately, the paradigm in which an overproduced tumor-specific protein can be easily detected as a marker of cancer has turned out to be the exception rather than the rule: in the nearly 160 years since Bence-Jones’ discovery, less than 10 proteins have progressed to the level of FDA-approved cancer diagnostic tests, and most of these lack ideal sensitivity and specificity for cancer.

In recent years “… the emerging science of genomics and proteomics have generated a plethora of candidate cancer biomarkers” (Pritzker 2002). Unfortunately few of these markers immediately stand out as superior prognostic or diagnostic tools, and even fewer have been validated and approved. Several factors might account for the slow pace of advance in cancer biomarkers. On the one hand, available proteomics technology has limited power to detect low-abundance cancer biomarkers against the background of high-abundance plasma proteins, and many of the best markers may thus be missed until discovery technology improves. On the other hand, the capacity to verify and validate existing candidate markers (through rigorous testing in large sample sets from many diseases) is limited, and it is therefore possible that the required biomarkers have already been “discovered” but not yet validated. In this paper, we are concerned with the latter possibility, and specifically with the problem of selecting among the existing candidates those that are most promising for systematic validation.

This line of enquiry immediately raises the question: where is the list of known candidate cancer biomarkers? While a number of useful reviews and books discuss specific cancer markers with clinical promise, these generally concentrate on proven, or at least well-developed, markers or specific disease states. We were unable to find a list that draws together a large population of candidates at all stages of development from multiple discovery sources, and thus our first step has been to create one through a combination of literature search and other methods.

The value of a list of existing candidates could be limited by the general lack of sensitivity and specificity exhibited by most of the cancer markers found to date, a factor that may have discouraged others from undertaking this task previously. Most candidates that have been followed up in larger studies have shown poor diagnostic value (Table 1), and even those that have been approved for clinical use exhibit lower sensitivity and specificity than the well-known markers of, eg, acute cardiovascular events (ie, troponin in myocardial infarction or B-type natriuretic peptide in congestive heart failure, Table 1).

On the other hand, there seems to be a growing consensus that panels of markers may be able to supply the specificity and sensitivity that individual markers lack. For example a panel combining four known biomarkers (leptin, prolactin, osteopontin, insulin-like growth factor II), none of which used alone could distinguish patients from the controls, achieved a sensitivity and specificity of 95% for the diagnosis of ovarian cancer (Mor et al. 2005). In this case a combination of known proteins in a novel panel provided a significant advance. Xiao et al. identified 299 proteins in tissue culture by 1-D page and nano-ESI-MS/MS but then used ELISA to test 13 of the most interesting in serum. They reported that CD98, fascin, the secreted chain of the polymeric immunoglobulin receptor and 14-3-3 eta provide greater sensitivity when used together as a panel than any of the markers used alone (Xiao et al. 2005).

If, as we and others (Conrads et al. 2003) believe, panels of proteins provide the most promising avenue towards early and accurate cancer detection, then a re-examination of known candidates provides a logical approach to panel generation, with the expectation that a stream of new markers can be added as they are identified by marker discovery studies. This candidate-based, or targeted, approach will require a comprehensive list of prioritized candidates coupled with a technology able to assay these in large sets of plasma and serum samples from clinical and epidemiological studies (together a “biomarker pipeline” (Anderson 2005b)).

Here we have begun to compile and prioritize a database of candidate biomarkers reported to be differentially expressed in studies of human cancer. We have included changes observed either at the protein (plasma or tissue) or nucleic acid (tissue DNA or RNA) level for any cancer, and excluded results restricted to animal, cell culture systems, or single case report studies in hopes of focusing on the most promising clinical biomarker candidates. We hypothesize that the protein version of most, if not all of these markers should be detectable in blood plasma at some level, irrespective of the tissue source, ultimately allowing for their use in patient screening, diagnosis or follow-up.

## Experimental Procedures

### Search strategy

The principal strategy for creation of our list involved compilation of designated cancer related proteins from: our previously published work (Anderson et al. 2004), PubMed literature searches, cancer microarrays (868 proteins from 111 human cancer Superarrays (http://www.superarry.com and in supplemental material), Circulating Tumor Markers of the New Millennium (Wu 2002), American Association for Clinical Chemistry abstracts and general literature perusal. PubMed searches included de novo PubMed literature searches: [plasma (Title/Abstract) NOT membrane (Title/Abstract) NOT stimulation (Title/Abstract) NOT drug (Title/Abstract) NOT dose (Title/Abstract) AND protein (Title/Abstract) AND cancer] and [“cancer antigen” AND human], as well as the PubMed literature search used for proteins from other sources [“protein name” AND cancer AND human AND (where necessary) diagnostic AND (where necessary) expression] and PubMed “related article” searches. Only proteins for which we found at least one published study on human cancer utilizing primary samples were retained (639 of the array proteins). Each biomarker reference was then manually tabulated and curated as to disease and tissue (including plasma). Single case studies were excluded.

### Clinical use data

FDA approval dates for tests were obtained from the FDA Center for Devices and Radiological Health database. Proteins designated here as clinical markers are those offered commercially by ARUP or by Mayo Medical Laboratories, or else offered for internal use by either NIH or the Fred Hutchinson Cancer Research Center.

### Citation analysis

Each documented protein on the resulting list was searched against the literature (via PubMed) using the query [“protein name” AND human AND cancer AND diagnostic]. This is admittedly a crude metric of research interest in a biomarker, but provides a useful method of relative prioritization among markers. In tabulating citation frequencies we did not exclude those categories ruled out in compiling the list initially: studies of animal systems, single clinical cases, or cell lines. If the “protein name” was not found by this search strategy it was counted as zero. It must be noted that PubMed is not a static archive but rather constantly changing both by additions, subtractions, and redefinition of MeSH headings. Still this exercise allowed some relative ranking of interest and therefore importance. Total cancer citations per year were determined using the query [human AND cancer AND diagnostic] limited to a specific publication year.

### Annotation

Swiss-Prot/Uniprot accession numbers were obtained where possible. Most of the TrEMBL annotations were done prior to the addition of species information to the annotation number and so this form of the annotation was maintained. Candidate cancer biomarkers were annotated with GO numbers and IDs from EBI’s human GOA 30.0 (gene_association.goa_human, ftp://ftp.ebi.ac.uk/pub/databases) and the Gene Ontology’s GO.def version 1.213 (http://www.geneontology.org/ontology/GO.defs) respectively. Similar ID groupings were then combined. The entire Human GO file was treated in an identical fashion for comparison with the candidates.

### Protein concentrations

Where possible, normal or control values for the plasma concentration of each protein were obtained by literature search. Unless specifically noted, protein concentrations are for the intact protein not individual subunits.

## Results

A search strategy combining literature search, extraction from microarray data, and a review of existing clinical tests, followed by manual curation, provided a list of 1261 candidate protein biomarkers (supplemental material) for which we found evidence of a quantitative change in some human cancer. As shown in Table 2, the candidates included proteins known to occur in plasma (274), proteins detected in tissue samples (542), and proteins whose corresponding mRNA or DNA levels were differentially expressed between cancerous and normal samples (656). These categories are non-exclusive in that a significant number of the candidates were found in more than one type of study. Proteins detected in the plasma represent 22% of the total proteins documented to date.

### Citation frequency

**Figure 1:**
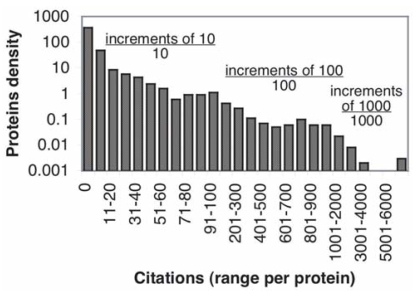
Biomarker Citation Frequency. Citation Frequency for each protein was determined using the PubMed query [“protein name” AND human AND cancer AND diagnostic]. Proteins were then histogrammed in bins of 10, 100 and 1000 citations (for frequencies of n<100, 100<n<1000, and n>1000, respectively) and each bin’s count normalized through division by bin size (eg the count of proteins falling in the 11–20 citations bin was divided by 10).

Citation frequency analysis was used as one method of prioritizing the biomarkers, on the assumption that proteins most widely studied in the context of cancer had more promise as biomarkers. Citation frequency was determined using a PubMed query intended to count citations in which the authors considered the proteins to have diagnostic value (Figure 1, Table 3). When this is done, 29% of the 1,261 biomarkers have no such citations, 67% have fewer than 10, and 74% fewer than 20. Likewise only a very limited number of biomarkers have extensive citations, 62 proteins or only 5% of the total number of biomarkers were found to have greater than 500 citations.

### Biomarkers with greater than 500 citations

**Figure 2:**
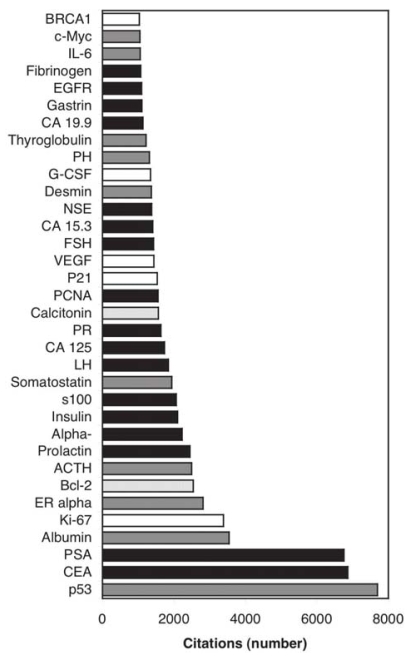
Proteins with greater than 1000 citations in Fig 1. White bars indicate non plasma proteins not used clinically, light gray bars indicate clinically used proteins not yet detected in plasma, dark gray bars indicate plasma proteins not used clinically and black bars indicate plasma proteins used clinically. CEA = Carcinoembryonic Antigen, PSA = Prostate Specific Antigen, ER alpha = Estrogen Receptor alpha, LH = Luteinizing Hormone, PR = Progesterone Receptor, PCNA = Proliferating Cell Nuclear Antigen, FSH = Follicle-stimulating Hormone, NSE = Neuron-specific enolase, PH=Parathyroid Hormone.

**Figure 3:**
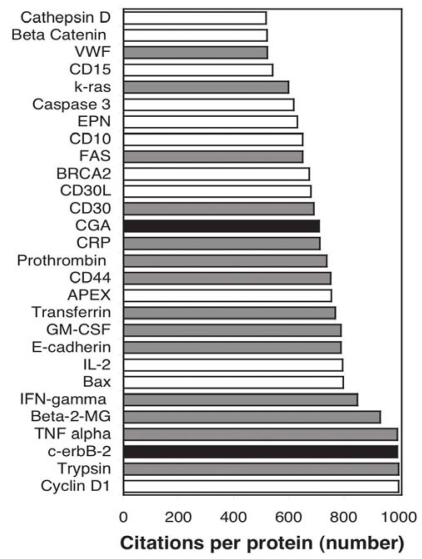
Proteins with greater than 500 but less than 1000 citations in Fig. 1. White bars indicate non-plasma proteins not used clinically, dark gray bars indicate plasma proteins not used clinically and black bars indicate plasma proteins used clinically. Beta-2-MG = Beta–2-microglobulin, IFN-gamma = IFN-gamma, CRP = C reactive protein, CGA = Chromogranin A, EPN = Erythropoietin, VWF=Von Willebrand Factor.

Of the 34 biomarkers with more than 1000 citations (Figure 2, Table 3) 79% are found in the plasma and 56% are presently used clinically (89% of which are reported in plasma). Of the 28 markers with between 500 and 1000 citations (Figure 3) 57% are plasma proteins but only 7% are used clinically. Both of the markers used clinically are plasma proteins. Some proteins with high citation frequency (eg, albumin) are somewhat surprising to see in the context of cancer biomarkers; these have been retained nevertheless because they appear to have reasonable relevance (low serum albumin levels are prognostic of poor survival (Lis et al. 2003) as noted in the table contents).

### Proteins with a large number or percentage of citations in 2004

**Figure 4:**
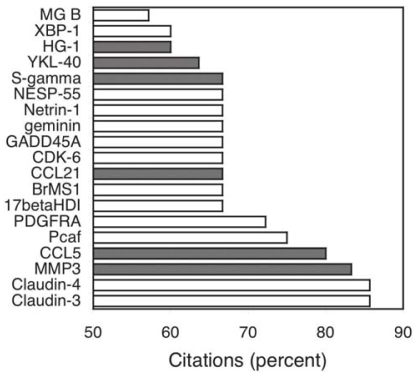
Proteins of “recent” interest (more than 50% of Fig. 1., citations occurring in 2004). White bars indicate non-plasma proteins not used clinically, dark gray bars indicate plasma proteins not used clinically. MG B = Mammaglobin B, HG = Haptoglobin 1, S-gamma = Synuclein-gamma, NESP-55 = Neuroendocrine secretory protein-55, CDK-6 = Cyclin-dependent kinase 6, 17betaHD1 = 17 beta-Hydroxys-teroid dehydrogenase type 1.

In an effort to include more recently discovered biomarkers we also looked at the proteins that had greater than 100 citations in 2004 or greater than 50% of their citations in 2004. Of the proteins with more than 100 citations in 2004, all but COX2 are represented in figures 2 and 3. Of those with a majority of total citations occurring in 2004, most have a low number (<10) of absolute citations (Figure 4, Table 3), 32% are detected in plasma and none are presently being used clinically.

### Time evolution of biomarker citations

**Figure 5:**
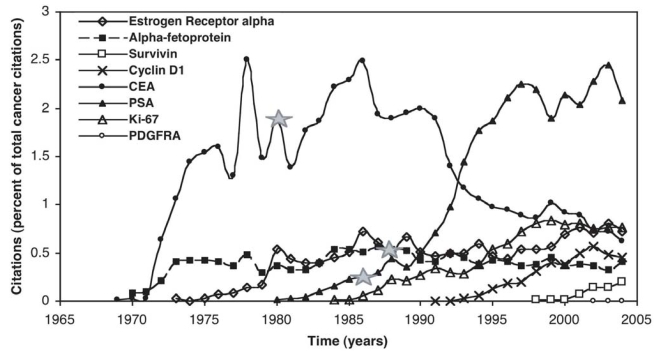
Evolution of Marker Interest. The number of times a marker is cited in a particular year divided by the total number of cancer citations for that year. Solid gray stars designate when the FDA approved CEA, PSA and alpha-fetoprotein. CEA = Carcinoembryonic Antigen, PSA = Prostate Specific Antigen, PDGRFR = Platelet-derived Growth Factor Receptor alpha.

We tracked the number of citations per year for selected cancer biomarkers over the last 35 years (Figure 5). The number of times a protein was cited in a given year (“protein name” AND cancer AND human AND diagnostic) was divided by the total number of cancer citations for that year (cancer AND human AND diagnostic) to give a rough index of the prominence of the biomarker in cancer research. Although frequently cited in the 1970’s and 1980’s, interest in CEA has dropped dramatically. The most cited marker in this group, PSA, has well-documented limitations as a diagnostic yet it continues to be cited either as the only option or as the biomarker upon which to improve. Interest in most of these biomarkers evolves in a fairly similar way: each appears to take a few years to be recognized, followed by gradually increasing interest over the following 15 to 20 years. Of these markers the FDA has approved only three as diagnostic cancer antigens: alpha-fetoprotein, CEA, and PSA (approved May 31, 1988, October 15, 1980 and February 25, 1986 respectively; Figure 5). To date only 6 additional markers have been approved by the FDA under the category of tumor associated antigens: CA 19-9 in May of 2002, Her2/Neu in September of 2000, CA 15.3 in February of 1981, bladder tumor marker in April of 1997, thyroglobulin in March of 1999 and CA 125 in July of 1987 (Table 4). None of these markers, used singly, has over 90% sensitivity and specificity. Although these numbers are for specific assays, they are representative of the general lack of specificity and sensitivity of the individual cancer markers currently available.

### Concentration range of cancer plasma biomarkers

**Figure 6:**
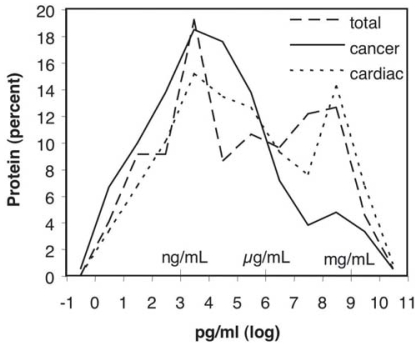
Distribution of Normal Plasma Concentrations for Plasma Cancer Biomarkers. The number of plasma concentrations falling within a given log of pg/ml were normalized to percent of total and then were histogrammed in log bins. The concentrations of the 211 cancer biomarkers detected in plasma are represented by the solid line, the concentrations of the unselected plasma proteins by the dashed line, and the concentration of cardiac biomarkers by the dotted line.

We attempted to collect normal plasma concentrations for candidate cancer biomarkers reported in the literature. The resulting 211 values were histogramed (Figure 6) for comparison with the distributions of concentrations of either unselected plasma proteins from PPI’s plasma protein database, or a set of candidate cardiovascular biomarkers (Anderson 2005a). The cancer candidates cover a >10-log concentration range with proteins such as immune modulating interleukins (1α andβ, 2, 5, 6, 9, 10, IFN-γ and GM-CSF) being present in normal plasma or serum in the pg/mL range while classical plasma proteins (albumin, transferrin, fibrinogen, and α-2-macroglobulin) are present at mg/mL levels. When the cancer candidate distribution is compared to the concentrations for all plasma proteins (unpublished results) and plasma markers of cardiac disease, a greater proportion of the cancer candidates appear in the lower concentration ranges than general plasma proteins or cardiac markers. Thus normal values for 185 (88%) of the markers for which we know the plasma concentration fall below 10 microgram/mL and 103 (49%) fall below 10 ng/mL. Tabulated concentrations are those found in controls not patients. Thus in many cases these may increase in cancer, thereby aiding in their detection.

### Genome Ontology classification of cancer candidate biomarkers

**Figure 7:**
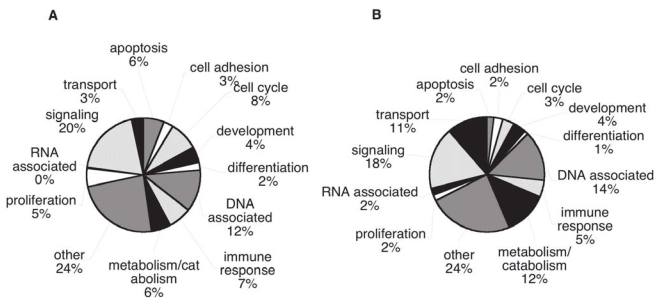
Distribution by Biological Process. Genome Ontology categories for A) Cancer biomarker proteins, B) Overall human proteome (genome data).

**Figure 8:**
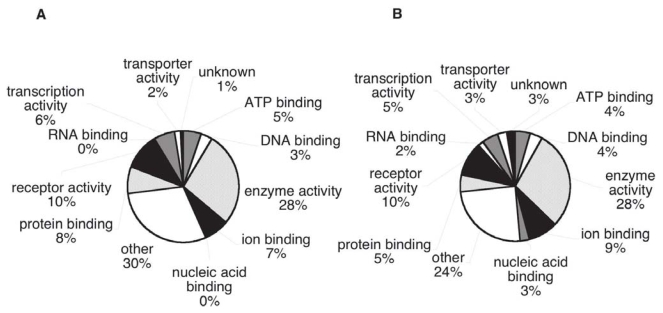
Distribution by Cellular Component. Genome Ontology categories for A) Cancer biomarker proteins, B) Overall human proteome (genome data).

We compared the distribution of GO annotations for the cancer candidates with the distribution for all annotated human proteins over a series of summary categories, with the aim of finding any large biases in the cancer group. In comparing “Biological Process” GO annotation, the cancer biomarkers show an increased representation of apoptosis, cell cycle and proliferation annotations; processes blocked or increased in tumors (Figure 7); while metabolism, catabolism and transport proteins are decreased. When the two sets are compared by “Cellular Component” GO terms (Figure 8), the extracellular category is over represented in the cancer biomarker database in comparison with the whole human database (20% versus 6% respectively). This is true even if the proteins found experimentally in plasma are excluded (12% extracellular). The other Cellular Component categories show only small differences between the proteins sets. Comparing “Molecular Function” GO terms, only small differences are apparent between the cancer candidates and the whole annotated human proteome.

### Prioritization of candidates

Given the size of the list of candidates resulting from our assembly procedure, we attempted to select a smaller subset of higher priority candidates as a starting point for consideration of assay development and clinical validation. This subset comprising 260 proteins (Table 3) was compiled from the most highly cited proteins, the “recent” markers, plasma proteins of known concentration (indicating existence of an assay) and any marker presently in any type of clinical use. Many of these markers fall into expected categories such as immune modulation molecules (acute phase proteins, coagulation factors, immune modulators); and mediators of classical cancer pathways (oncoproteins, angiogenic or apoptosis factors, tumor suppressors or antigens, cellular homing or proliferation molecules). Somewhat less expected perhaps is that almost 22 (8%) of these top 262 proteins are involved in hormonal action.

### Existence of a specific antibody

For each of the 260 high priority candidates, we performed web searches, primarily through the Exact Antigen website (www.exactantigen.com), to determine whether an antibody with potential utility in a plasma assay is commercially available. Relevant antibodies were found for 186 (72%) of the 260 high priority candidates.

## Discussion

According to the Centers for Disease Control, 1 in every 4 deaths in the United States is due to cancer. Many of these deaths could be averted by improved early cancer detection, since existing therapies, especially surgery, are much more effective in early cancer stages as compared to later stages (Etzioni et al. 2003). Billions of dollars have been spent on basic research looking for molecular differences related to cancer-work that has been at least partly motivated by the need for improved in vitro diagnostic tests to detect or monitor progression of cancer. Yet to our knowledge no centralized database of known candidate cancer biomarkers exists. Such a list could serve to confirm new results, eg, from proteomic comparisons of cancer and control sera, by placing them in a context of earlier work. Additionally it could serve as a reservoir of current and future candidates to be tested in large sample sets by candidate-based (“targeted” or “directed”) proteomics methods. The latter use is important, since candidate-based methods, consisting of specific assays for defined targets, are likely to be much more sensitive than proteome profiling methods, and hence could cover a much broader universe of protein candidates and potentially detect disease states earlier.

The present catalog of 1261 human candidate cancer biomarkers is a first attempt at such a database. We did not select specific cancer types or specific detection methods, choosing instead to cast a broad net. In the resulting list, it will be apparent that the strength of evidence and likelihood of ultimate usefulness of the candidates varies widely. Even candidates that have been tested and found to have poor diagnostic specificity and sensitivity were retained, as they may nevertheless contribute to useful panels as in the work of Mor and Xiao. Looking at the list, one might question why the most abundant plasma protein (serum albumin) is included – though perhaps counter-intuitive, albumin does meet the search criteria used, and is in fact a useful negative acute phase indicator likely to be altered in cancer along with many inflammation-related proteins. Other well-known proteins not usually considered as cancer-specific are also included (eg, protein and peptide hormones overproduced by endocrine tumors or through ectopic synthesis). Overall, the list is not easily recognizable by inspection as a list of cancer markers.

Of the 1261 proteins, 22% are reported to occur in plasma. This is an appreciable fraction considering that many of the large array studies, capable of finding many markers per experiment, have looked for differential protein or DNA expression in tissues. For bona fide cell-associated cancer markers such as Her-2, there is persuasive evidence that at least a fragment of the protein molecule is released into the plasma and can be detected as a cancer biomarker (Tse et al. 2005), and other proteins documented here in the tissues of cancer patients have been demonstrated to be found in plasma in other disease indications. These cases provide some support for the hypothesis that most if not all of the 1261 proteins should be detectable at some level in plasma, the diagnostic sample of choice, given a sensitive enough assay. Whether current assay technologies will be sensitive enough to see a large fraction of the candidates in plasma is a major question at this point, and one that will require vigorous efforts to resolve.

As might be expected, there is a smooth distribution in the number of literature citations per candidate, ranging from almost 8,000 (for PSA) to zero (for candidates not mentioned as diagnostic by the publication’s authors). This result suggests that our literature analysis did not identify a crisply defined set of cancer markers, but rather part of a continuum extending from a few established markers through plausible candidates into more speculative possibilities. Given the complexity of cancer, such an outcome is not surprising.

Only 5% of the 1261 candidates have been extensively studied (500 or greater total citations over the years). When examined as a function of time, the citation history of individual markers appears to show a slow evolution of interest that peaks 15 to 20 years after the initial papers. Only in the cases of CEA and PSA was discovery of a biomarker followed by a rapid increase in publications over a few years and in the case of PSA the steady increase was seen only 10 years after the first citations appeared. Thus in order to catch recently emerged candidates, we focused on candidates with a high proportion of citations occurring in 2004 but with fewer total citations (often 10 or less). Of the total 1261 proteins only 41 are used in some clinical sense and even fewer have FDA approved assays.

While the observed slow pace is easily explained by the deliberate nature of clinical research and the progressive, rather than abrupt, nature of adoption in medical practice, it presents a stark reminder of the challenge involved in making any rapid advance in cancer diagnostics.

These candidate cancer markers, taken as a group, appear to be present in plasma at lower concentrations than comparable groups of cardiac markers or unselected plasma proteins. Although systematic biases in selection of these groups could affect this result, it tends to support the contention that plasma cancer marker discovery is, and may continue to be, a challenge in terms of detection sensitivity. Present discovery proteomics platforms typically detect proteins with plasma concentrations in the mg/mL to microg/mL range. For the proteins in our list with known plasma concentrations, we estimate that 86% would be missed by most conventional proteomics platforms, while 48% would be missed by high-end proteomics platforms with extensive multi-dimensional fractionation. For the present, the only way that many of these proteins can be detected is by specific assays: ie, by targeted proteomics. Targeted proteomics thus represents a preferred path to validation and further study of the candidate markers listed here.

The distribution of our cancer biomarker candidate proteins among GO annotation categories shows remarkable similarity to the distribution for all annotated human proteins. There is some enrichment for proteins annotated as related to apoptosis, cell cycle and proliferation (in the GO biological process category), as would be expected on account of the fundamental involvement of these processes in cancer. The extracellular group (in the GO cell component category) is also somewhat over-represented, a trend favorable to detection in plasma. Nevertheless the candidates seem to represent a very wide sampling of the human proteome.

The full set of these 1,261 candidates is too large to submit for immediate verification and validation in large sample sets by any available means, and some method of prioritization is required to initiate their evaluation. As an initial approach, we have selected a subset of the candidates based on a set of criteria including number of total citations, number of recent citations, proportion of recent citations, known plasma concentration (implying existence of an assay) and clinical use in any context. This subset of 260 candidates (presented in Table 3) includes 186 candidates for which a relevant antibody is commercially available, opening the possibility of testing this group using an antibody array or other miniaturized immunoassay technology in the near future.

While the list of candidate cancer biomarkers assembled here is clearly a simplistic and therefore somewhat crude initial catalog, we believe the result will prove to be of sufficient value to justify extending the effort to provide an ongoing summary of the progress of cancer diagnostics. In particular we believe that linking a database of marker candidates to the bioinformatics architecture used in biomarker discovery will help to connect the discovery and validation phases (Anderson 2005b) necessary for progression of biomarkers to the clinic. One can envision a steady accumulation of candidates, regular revision of candidate priorities as evidence emerges from multiple sources (literature, microarrays, systems models, etc), and finally feedback in the form of specific measurements from validation studies in large sample sets. Such a collection of data would provide an up-to-date snapshot of the workings of a cancer diagnostic marker pipeline.

Finally, lists such as this prompt important, but infrequently-asked questions regarding the most productive tack for future discovery efforts. Is it reassuring to find confirmation of fresh observations through overlap with a pre-existing list? Perhaps so, and particularly if the candidates involved appear repeatedly in similar independent studies. However the sieve used here is crude and so our list cannot really “confirm” a candidate seen in a new study–overlap just improves the odds of relevance. Further, since there are certain to be good cancer markers not on this list, failure to appear here in no way disqualifies a novel marker. Hence our hope is to contribute a mechanism for marginally improving chances of recognizing a valid marker, and a systematic source for enriched candidates available for validation and panel assembly efforts.

## Figures and Tables

**Figure 9:**
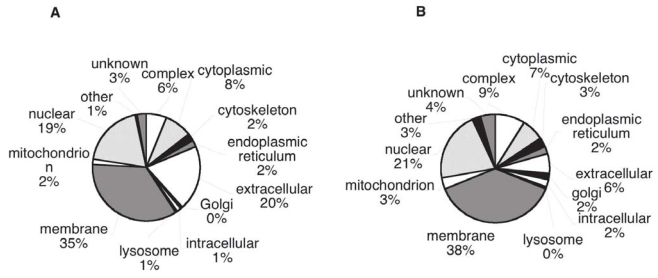
Distribution by Molecular function. Genome Ontology categories for A) Cancer biomarker proteins, B) Overall human proteome (genome data).

**Table 1:** Example sensitivities and specificities for the nine FDA approved cancer biomarkers.

Marker	Disease	Cut Off	Sensitivity	Specificity	Reference
CEA	malignant pleural effusion	NA^1^	57.5%	78.6%	(Li et al. 2003)
CEA	peritoneal cancer dissemination	0.5 ng/ml	75.8%	90.8%	(Yamamto et al. 2004)
Her-2/neu	stage IV breast cancer	15 ng/mL	40%	98%^2^	(Cook et al. 2001)
Bladder Tumor Antigen	urothelial cell carcinoma	NA	52.8%	70%	(Mian et al. 2000)
Thyroglobulin	thyroid cancer metastasis	2.3 ng/ml^3^	74.5%	95%	(Lima et al. 2002)
Alpha-fetoprotein	hepatocellular carcinoma	20 ng/ml	50%	70%	(De Masi et al. 2005)
PSA	prostate cancer	4.0 ng/mL	46%	91%	(Gann et al. 1995)
CA 125	non-small cell lung cancer	95 IU/mL	84%	80%	(Dabrowska et al. 2004)
CA19.9	pancreatic cancer	NA	75%	80%	(Yamaguchi et al. 2004)
CA 15.3	breast cancer	40 U/ml	58.2%	96.0%	(Ciambellotti et al. 1993)
leptin, prolactin, osteopontin, and IGF-II	ovarian cancer	NA	95%	95%	(Mor et al. 2005)
CD98, fascin, sPIgR^4^, and 14-3-3 eta	lung cancer	NA	96%	77%	(Xiao et al. 2005)
Troponin I	myocardial infarction	0.1 microg/L	93%	81%	(Eggers et al. 2004)
B-type natriuretic peptide	Congestive heart failure	8 pg/mL	98%	92%	(Dao et al. 2001)

1 Not available

2 vs benign breast diseases

3 vs 3rd week post surgery

4 Secreted chain of the polymeric immunoglobulin receptor

**Table 2:** Distribution of cancer biomarkers. Other = amniotic, bile, cerebrospinal fluid, follicular fluid, milk of lactating women, pancreatic fluid, seminal plasma, sputum, stools and urine.

1261 Unique proteins	Proteins in plasma	Tissue proteins	DNA & RNA data	Other
Proteins in plasma	274	60	24	6
Tissue proteins	60	542	152	6
DNA & RNA data	24	152	656	3
Other	6	6	3	15

**Table 3:** High priority cancer markers. Proteins having > 500 total citations, >100 citations in 2004, >50% 2004 citations, a known plasma concentration or used clinically are listed.

Protein Names	Citations			Plasma Conc Known in pg/ml	Clinical Markers	Total # of ✓ s	Available Antibody	Human Swiss Prot #	Control Plasma conc pg/ml	Concentration Reference	Comments
	Total >500	2004 >100	2004/Total x100 >50								
Alpha-fetoprotein	✓	✓		✓	✓	4	yes	P02771	2.0E+04	(Beneduce et al. 2004)	A pregnancy associated oncofetal protein reexpressed in hepatocellular cancer, cirrhosis and hepatitis (Cheema et al. 2004).
Carcinoembryonic antigen	✓	✓		✓	✓	4	yes	P06731	1.0E+03	(Mavligit and Estrov 2000) (Engaras et al. 1999)	A reexpressed onco-fetal protein, CEA is currently in use in colorectal cancer diagnosis even though its sensitivity can be particularly low in the initial stages (Heptner et al. 1984).
Epidermal growth factor receptor	✓	✓		✓	✓	4	yes	P00533	6.9E+06	(Baron et al. 2001)	A membrane tyrosine kinase that inhibits apoptosis and promotes angiogenesis. Found to be connected with increased malignancy (Jeziorski et al. 2000).
Kallikrein 3 (prostate specific antigen)	✓	✓		✓	✓	4	yes	P07288	1.9E+03	(Herrmann et al. 2004) (Ng et al. 2005)	PSA hydrolyzes the high molecular mass seminal vesicle protein thus leading to the liquid fraction of the seminal coagulum It is increased in men with prostate cancer (Thakur et al. 2003).
Vascular endothelial growth factor A, VEGF	✓	✓		✓	✓	4	yes	P15692	2.0E+02	(Miyashita et al. 2003)	VEGF is a potent angiogenic factor. Serum levels have been detected in melanoma (Ugurel et al. 2001), pituitary (Komorowski et al. 2000) and colorectal carcinomas (Davies et al. 2000).
Albumin	✓	✓		✓		3	yes	P02768	4.1E+10	(Laboratories 2001)	A serum protein responsible for colloidal osmotic pressure and plasma molecule transport. It is decreased in end stage renal disease(Kaysen and Kumar 2003).
CA 125	✓	✓			✓	3	yes	x		(Woolas et al. 1993) (Hasholzner et al. 1994)	A monoclonal antibody identified cancer antigen that is Elevated in most clinically advanced ovarian carcinomas and which may be elevated prediagnosis CA 125 is a potentially useful for early detection. However, CA 125 is not always elevated in malignant cancer and can be elevated in benign ovarian tumors (McIntosh et al. 2004).
Calcitonin	✓			✓	✓	3	yes	P01258	1.0E+01	(Karanikas et al. 2004)	A thyroid hormone that lowers calcium and phosphate levels and inhibits bone resorption, calcitonin is useful in the detection of thyroid cancers however it is also elevated in Hashimoto’s thyroiditis (Karanikas et al. 2004).
Chromogranin A (parathyroid secretory protein 1)	✓			✓	✓	3	NF	P10645	6.5E+04	(Pujol et al. 2003)	A neuroendocrine secretory protein secreted by tumours with neuroendocrine properties. The assay is used primarily in the diagnosis and monitoring of patients with tumours of neuroendocrine origin. Increased levels in small cell lung cancer patients are associated with shorter survival (Pujol et al. 2003).
Corticotropin-lipotropin contains ACTH	✓			✓	✓	3	yes	P01189	1.1E+01	(Walsh et al. 2005)	Coritcotropin-lipotropin contains melanotropin which increases pigmentation of the skin and ACTH which stimulates the adrenal glands to secret cortisol. It is secreted by some pituitary tumors (Chanson and Salenave 2004). Concentration for ACTH.
Estrogen receptor 1	✓	✓			✓	3	yes	P03372			The estrogen receptor is a steroid receptor which stimulates hormone-specific transcription of specific genes. Most breast cancers express estrogen and progesterone receptor (ERalpha and PR) (Clarke et al. 2005).
Gastrin	✓			✓	✓	3	yes	P01350	6.9E+02	(Triantafillidis et al. 2003)	A hormone that stimulates HCl secretion by the gastric mucosa, it is increased in gastric and colorectal cancer patients (Triantafillidis et al. 2003).
Progesterone receptor	✓	✓			✓	3	yes	P06401			The progesterone receptor is a steroid receptor which stimulates hormone-specific transcription of specific genes. There is a loss of expression in prostate cancer tissue (Ji et al. 2005).
Prolactin	✓			✓	✓	3	yes	P01236	1.6E+04	(Al Sifri and Raef 2004)	A hormone that stimulates and sustains lactation Multiple regression analysis showed a significant correlation between tumor volume and serum PRL level in prolactinoma (Ma et al. 2002).
S100 alpha chain	✓	✓		✓		3	yes	P23297	9.0E+01	(Tas et al. 2004)	A calcium binding protein, S100 has been described as a useful tumor marker for malignant melanoma (Tas et al. 2004). Concentration is for the complex protein.
Somatostatin	✓			✓	✓	3	NF	P61278	2.0E+01	(Neradilova et al. 1989)	Somatostatin inhibits secretion of growth hormone, insulin, glucagon, gastrin , cholecystokinin, secretin and vasoactive intestinal peptide among others. It has been detected in the sera of 14–15% of lung cancer patients although tumor cell expression appears rare (O’Byrne et al. 2001).
Thyroglobulin	✓			✓	✓	3	yes	P01266	1.0E+03	(Montella et al. 2004)	Precursor to the thyroid hormones thyroxine and triiodothyronine its level in plasma is used in the management of thyroid cancer (Whitley and Ain 2004).
V-erb-b2, Her2/neu	✓			✓	✓	3	yes	P04626	1.1E+04	(Wu 2002)	An oncogene product whose tissue expression and levels of the shed protein in serum have been shown to correlate with tumor stage in a range of adenocarcinomas (Tsigris et al. 2002).
Antigen identified by monoclonal antibody Ki-67	✓	✓				2	NF	P46013			A proliferation-associated antigen that is increased in small cell lung cancer patients (Grefte et al. 2004).
B-cell CLL/lymphoma 2	✓	✓				2	yes	P10415			An inhibitor of apoptosis Bcl-2 maintains homeostasis in the immune system The differing effects of Bcl-2 expression on prognosis may be due to which cells are expressing the Bcl-2, immune cells or tumors. High expression in ovarian cancer (Herod et al. 1996) and non small lung cancer (Shibata et al. 2004) are associated with better prognosis whereas well differentiated tumors more likely to be Bcl-2 positive (Soda et al. 1999).
BCL2-associated X protein	✓	✓				2	yes	Q07812 Q07814 Q07815			Bax is an apoptosis inhibitor highly expressed in Hodgkin’s disease (Schlaifer et al. 1995).
Beta-2-microglobulin	✓			✓		2	yes	P61769	2.1E+06	(Bien et al. 2004)	The nonpolymorphic chain of MHC class I molecules. It is slightly increased in children with acute leukemias and lymphomas but not in solid tumor disorders.
Breast cancer 1 early onset	✓	✓				2	yes	P38398			The BRCA1 protein is a tumor suppressor that mediates DNA damage and repair, transcriptional activity and chromosomal stability. However, while inherited mutations of BRCA1 are responsible for about 40–45% of hereditary breast cancers, these mutations account for only 2–3% of all breast cancers (Rosen et al. 2003).
CA 15.3	✓				✓	2	yes	x			A monoclonal antibody identified cancer antigen increased in patients with metastatic breast cancer (Lockhart et al. 1999).
CA 19.9	✓				✓	2	NF	x			A monoclonal antibody identified cancer antigen increased in colorectal cancer patients (Lockhart et al. 1999).
Cadherin 1 type 1 E-cadherin (epithelial)	✓			✓		2	yes	P12830	7.0E+06	(Chan et al. 2001)	E-cadherin, a cell adhesion protein, plays a role in tissue formation and architecture. Elevated levels of sE-cadherin are found in sera of patients with bladder cancer and correlate with known prognostic factors. (Griffiths et al. 1996).
Caspase 3	✓	✓				2	yes	P42574			Caspase 3 is involved in not only apoptosis execution but also proliferation. It has been shown to be downregulated in gastric lymphoma tissue but negatively associated with lymph node metastases in gastric carcinoma (Sun et al. 2004).
CD44 antigen	✓			✓		2	yes	P16070	2.2E+05	(Lockhart et al. 1999)	Certain CD44 isoforms that regulate activation and migration of lymphocytes and macrophages may also enhance local growth and metastatic spread of tumor cells. Present in serum of normal individuals it is elevated in the serum from gastric and colon cancer patients, (Guo et al. 1994), Hodgkin’s lymphoma patients(Lockhart et al. 1999), and acute leukemia patients (Yokota et al. 1999).
Cellular tumor antigen p53	✓	✓				2	yes	P04637			The p53 tumor suppressor protein regulates proliferation, cell cycle checkpoints, and apoptosis. More than one half of all lung cancers contain a mutation of the p53 tumor suppressor gene (Johnson and Kelley 1993).
Coagulation factor II, prothrombin	✓			✓		2	yes	P00734	1.2E+03	(McKenzie et al. 1999) conc. for thrombin fragment	A coagulation factor seen in cancers with deep venous thrombosis (Goldenberg et al. 2003).
Colony stimulating factor 2 (granulocyte-macrophage)	✓			✓		2	yes	P04141	1.0E+01	(Suzuki et al. 1992)	A hematopoietic cytokine that promotes the maturation of bone marrow cells into antigen presenting cells. Some metastatic tumors produce GM-CSF (Suzuki et al. 1992).
Colony stimulating factor 3 (granulocyte)	✓			✓		2	yes	P09919	1.8E+01	(Ishida et al. 2004)	G-CSF is a hematopoietic cytokine generated at infection sites to recruit and replace neutrophils consumed in an immune reaction. It is produced by some metastatic tumors (Suzuki et al. 1992).
C-reactive protein	✓			✓		2	yes	P02741	2.0E+06	(Bolayirli et al. 2004)	An inflammation indicator, increased CRP levels are considered to be an important risk factor for atherosclerosis, myocardial infarction, peripheral vascular disease, and ischemic stroke. It is positively correlated with weight loss, anorexia-cachexia syndrome, extent of disease, and recurrence in advanced cancer. Its role as a predictor of survival has been shown in multiple myeloma, melanoma, lymphoma, ovarian, renal, pancreatic, and gastrointestinal tumors (Mahmoud and Rivera 2002).
Cyclin D1	✓	✓				2	yes	P24385			Cyclins are in all proliferating cell types and collectively control the progression of cells through the cell cycle. Genetic alterations affecting p16(INK4a) and cyclin D1, proteins that govern phosphorylation of the retinoblastoma protein (RB) and control exit from the G1 phase of the cell cycle, are so frequent in human cancers that inactivation of this pathway may well be necessary for tumor development (Sherr 1996).
Cyclin-dependent kinase inhibitor 1, p21	✓	✓				2	yes	P38936			P21 is a cyclin-dependent kinase inhibitor that blocks cell cycle progression. It is suppressed in malignant nasopharyngeal epithelial cells(Fung et al. 2000), but overexpressed in pancreatic ductal adenocarcinoma (Hermanova et al. 2004).
Erythropoietin	✓			✓		2	yes	P01588	1.0E+05	(Masaki et al. 1992)	A stimulator of erythropoiesis associated with malignant cells and tumor vasculature in breast cancer (Acs et al. 2001).
Fibrinogen alpha/alpha-E chain	✓			✓		2	yes	P02671	2.7E+09	(Bolayirli et al. 2004)	A coagulation factor increased in cancer patients without inflammation (Bolayirli et al. 2004).
Follicle-stimulating hormone	✓				✓	2	yes	P01225			Follicle-stimulating hormone enables ovarian folliculogenesis to the antral follicle stage and is essential for Sertoli cell proliferation and maintenance of sperm quality in the testis. It is decreased in testicular cancer (Madersbacher et al. 1998).
Gamma enolase	✓			✓		2	yes	P09104	1.3E+04	(Barlesi et al. 2004)	Neuron specific enolase, a glycolytic enzyme, is released into the CSF when neural tissue is injured. Neoplasms derived from neural or neuro-endocrine tissue may release NSE into the blood. Elevated levels are found in seminomas (Fossa et al. 1992), advanced non-small cell lung cancer (Barlesi et al. 2004), solid malignant tumors and malignant hematologic disorders (Burghuber et al. 1990).
Insulin	✓				✓	2	yes	P01308			Serum insulin levels were clearly higher in patients with breast cancer than in patients with benign breast disease and healthy controls (Han et al. 2005).
Interferon gamma	✓			✓		2	yes	P01579	1.0E+01	(Arca et al. 2004)	An inflammatory cytokine decreased in squamous cell carcinoma of the head and neck (Lathers et al. 2003).
Interleukin 2	✓			✓		2	yes	P60568	5.0E-01	(Lathers et al. 2003)	A T cell growth factor with roles in the specific immune system, expression of IL-2 is high in infiltrative breast tumors (Garcia-Tunnon et al. 2004).
Interleukin 6	✓			✓		2	yes	P05231	5.0E+00	(Lathers et al. 2003)	IL-6 is a cytokine that activates both innate and specific immune pathways. It is present in patients with metastatic renal (Walther et al. 1998), prostate (Adler et al. 1999), oral cavity and oropharyngeal squamous cell carcinoma (St. John et al. 2004).
k-ras	✓			✓		2	yes	P01116	1.7E+02	(Tsao et al. 2004)	An oncogene product found in approximately 90% of human pancreatic cancer (Sakuma et al. 2004). 22.5% of ovarian cancers expressed K-ras codon 12 point mutations (Semczuk et al. 2004).
Neprilysin, CD10	✓			✓		2	yes	P08473	2.5E+02	(Zhang et al. 1994)	CD10 is a B cell linage marker demonstrated to be positive in endometrial stromal sarcoma (Mikami et al. 2002).
Transferrin	✓			✓		2	yes	P02787	4.0E+09	(Stevens et al. 1986)	A serum iron transporter found to be decreased in laryngeal cancer (Taysi et al. 2003).
Trypsin	✓			✓		2	yes	P07477	9.9E+04	(Adrian et al. 1979)	A hydrolytic enzyme whose activity was significantly lower in hepatocellular cancer tissue (Niewczas et al. 2002) but not altered in pancreatic, stomach, colon, rectal, lung or breast adenocarcinomas.
Tumor necrosis factor (TNF-alpha)	✓			✓		2	yes	P01375	5.9E+00	(Straczkows ki et al. 2002)	TNF-alpha is a proinflammatory protein detected in the serum of 36.5% of pancreatic cancer patients. Patients with metastatic disease showed significantly higher positive serum TNF-alpha compared to those with non-metastatic disease (Karayiannakis et al. 2001).
Tumor necrosis factor receptor superfamily member 6, fas	✓			✓		2	yes	P25445	1.5E+03	(Hefler et al. 2000)	An apoptosis death receptor whose soluble form has been shown to be increased in serum from ovarian (Hefler et al. 2000), hepatocellular (Sacco et al. 2000), bladder (Mizutani et al. 1998), and colon cancer patients (Kushlinskii et al. 2001).
Von Willebrand Factor	✓			✓		2	yes	P04275	1.1E+05	(Byrne et al. 2000)	A coagulation factor that reflects endothelial damage (Takatsuka et al. 1998), it is elevated in patients with colorectal cancer (Damin et al. 2002).
Chemokine ligand 5 (CCL5)			✓	✓		2	yes	P13501	3.7E+04	(Baer et al. 2005)	A CC chemokine involved in both cellular and humoral immunity. It is expressed by leukemic cells in peripheral blood and lymph nodes from patients with adult T-cell leukemia, an HTLV-I associated disease (Mori et al. 2004).
Chitinase-3 like protein 1, YKL-40			✓	✓		2	NF	P36222	2.8E+04	(Dupont et al. 2004)	YKL-40 (cartilage gp-39), is a mammalian glycoprotein related in sequence to chitinases. Its function is unknown, but it is thought to be involved in tissue remodeling (De Ceuninck et al. 2001). YKL-40 may represent a novel marker for the detection of early-stage ovarian cancer (Dupont et al. 2004).
Choriogonadotropin beta chain				✓	✓	2	yes	P01233	1.0E+02	<1.08 microg/ L, total protein (Rohsig et al. 2001) beta chain (Marcillac et al. 1992)	The beta chain of choriogonadotropin supports pregnancy and can be seen in gestational trophoblastic disease, gestational trophoblastic neoplasm, choriocarcinoma and placental site tumor cases as well as in testicular cancer and germ cell tumor (Cole and Sutton 2004).
Colony stimulating factor 1 (macrophage)				✓	✓	2	yes	P09603	7.0E+01	(Woolas et al. 1993)	A modulator that increases production of inflammatory leukocytes from the bone marrow, it is increased in ovarian cancer (Skates et al. 2004).
Haptoglobin-1			✓	✓		2	yes	P00738	1.3E+09	(Bolayirli et al. 2004)	It binds hemoglobin and is increased in conditions with extensive tissue damage and necrosis. It is increased in leukemia patients (Kwak et al. 2004). High levels in small cell lung cancer are associated with decreased survival (Bharti et al. 2004).
Hepatocyte growth factor				✓	✓	2	yes	P14210	2.0E+02	(Matsumori et al. 2000)	A growth factor for a broad spectrum of tissues and cell types. Hepatocyte growth factor has no detectable protease activity. It is increased in breast cancer tissues (Parr et al. 2004).
Inhibin				✓	✓	2	yes	various	3.0E+03	(Khosravi et al. 2004)	A glycoprotein hormone which regulates pituitary FSH, it is increased in postmenopausal ovarian cancer patients (Khosravi et al. 2004).
Interferon-alpha/beta receptor alpha chain			✓	✓		2	yes	P17181	1.7E+03	(Kanayama et al. 2000)	IFN receptor activation inhibits viral replication. In increasing order, higher levels are seen in benign hypertrophy, urolithiasis, bladder cancer , renal cell carcinoma, and prostate cancer (Kanayama et al. 2000). Concentrations for the complexed receptor.
Interferon-alpha/beta receptor beta chain			✓	✓		2	yes	P48551	1.7E+03	receptor (Kanayama et al. 2000)	IFN receptor activation inhibits viral replication. In increasing order, higher levels are seen in benign hypertrophy, urolithiasis, bladder cancer, renal cell carcinoma, and prostate cancer (Kanayama et al. 2000). Concentrations for the complexed receptor.
Kallikrein 10				✓	✓	2	yes	O43240	4.4E+02	(Luo et al. 2003)	Kallikrein 10 suppresses breast and prostate cancer. It is increased in tissues and serum of patients with ovarian cancer (Yousef and Diamandis 2002).
Kallikrein 11				✓	✓	2	yes	Q9UBX7	2.2E+06	(Diamandis et al. 2002)	A serine protease that may be involved in tissue remodeling and cell migration, it is elevated in ovarian cancer (Yousef et al. 2003).
Kallikrein 6				✓	✓	2	yes	Q92876	2.9E+03	(Diamandis et al. 2003)	A serine protease that may be useful in the diagnosis and monitoring of ovarian and prostate cancer (Yousef and Diamandis 2002). Increased plasma levels are also present in Alzheimer’s disease (Diamandis et al. 2000).
Matrix metalloproteinase 3			✓	✓		2	yes	P08254	8.0E+03	(Sangiorgi et al. 2001)	A secreted proteoglycanase produced predominantly by connective tissue cells. MMPs are capable of disintegrating the basement membrane, which is a main characteristic of tumor invasion. MMP3 is elevated in squamous cell carcinomas of the head and neck. Additionally MMP3 is not changed in inflammatory diseases (Kuropkat et al. 2002).
Small inducible cytokine A21 (CCL21)			✓	✓		2	yes	O00585	1.7E+02	R&D	CCL21 inhibits hematopoiesis and stimulates chemotaxis. It is differentially expressed in ovarian cancer (Mor et al. 2005).
soluble IL-2R alpha				✓	✓	2	yes	P01589	1.4E+03	(Beguin et al. 1993)	The IL-2 receptor is required for T cell activation. The preoperative levels of serum soluble IL-2R in patients with colorectal cancer were significantly higher than those of normal controls. The levels of serum soluble IL-2R in patients with metastatic lymph nodes were also significantly higher than the levels in those without metastatic lymph nodes (Sakata et al. 2002).
Somatotropin growth factor, growth hormone				✓	✓	2	yes	P01241 P01242	4.0E+02	(Krassas et al. 2003)	Somatotropin controls growth. T is increased in gastrointestinal cancer patients (Dulger et al. 2004).
Breast cancer 2 early onset	✓					1	yes	P51587			BRCA2 is a breast cancer susceptibility gene. Five percent of early onset breast cancer cases express mutations in Brca 1 or 2 (Lalloo and Evans 1999).
Catenin Beta 1	✓					1	yes	P35222			Beta-catenin is necessary for the establishment and maintenance of epithelial layers. Accumulated cytoplasmic beta-catenin has been seen in esophageal squamous cell carcinoma (Zhou et al. 2004).
Cathepsin D	✓					1	yes	P07339			A lysosomal proteinase, cathepsin D was found to be statistically significantly higher in colorectal cancer (Guzinska-Ustymowicz et al. 2004).
CD15	✓					1	yes	x			CD15 is a myeloid-associated surface antigen expressed on some myelomatous B cells and may be related to a poor prognosis (Ruiz-Arguelles and San Miguel 1994).
Desmin	✓					1	yes	P17661			Desmin is a muscle-specific cytoskeletal protein found in smooth, cardiac, and heart muscles. 855 of mesothelial hyperplasias showed desmin immunoreactivity (Attanoos et al. 2003).
DNA-(apurinic or apyrimidinic site) lyase, APEX	✓					1	yes	P27695			A DNA repair enzyme, increased immuno histochemical staining seen in prostate cancer tissue (Kelley et al. 2001).
Lutropin beta chain, Luteinizing hormone	✓					1	yes	P01229			A gonadotropic hormone decreased in breast cancer (Micheli et al. 2004).
Parathyroid Hormone	✓					1	yes	P01270			Stimulates bone formation A correlation between tumor activity and ACTH, CT and PTH levels was shown in 50.44 and 47% of lung cancer patients respectively (Ausekar et al. 1985).
Proliferating cell nuclear antigen	✓					1	yes	P12004			A DNA repair protein. Increased levels in breast cancer (Kushlinskii et al. 2004).
Tumor necrosis factor ligand superfamily member 8 (CD30 ligand)	✓					1	yes	P32971			CD30L is a cell surface activation antigen on monocytes, T and B cells; and constitutively expressed on granulocytes and medullary thymic epithelial cells. It is expressed in thyroid cancer tissue (Trovato et al. 2001).
V-myc myelocytomatosis viral oncogene homolog (avian)	✓					1	yes	P01106			An oncogene whose increased transcriptional activity is a characteristic feature of Burkitt’s lymphoma (Wilda et al. 2004).
Tumor necrosis factor ligand superfamily member 8 (CD30)	✓					1	yes	P28908			A lymphoid activation antigen overexpressed in Hodgkin’s disease (Horie et al. 2002).
17beta- Hydroxysteroid dehydrogenase type 1 (17HSD1)			✓			1	NF	x			17HSD1 converts estrone to estradiol in the ovary, placenta and the breast. Signals for 17HSD1 mRNA were detected in 16% of breast cancer specimens (Oduwole et al. 2004).
Acid phosphatase prostate				✓		1	yes	P15309	3.5E+03	(Laboratories 2001)	An enzyme produced by the prostate, it is increased in men with prostate cancer (Afzal et al. 2003).
Adrenomedullin				✓		1	yes	P35318	7.4E+01	(Ehlenz et al. 1997)	Adrenomedullin is a hypotensive peptide increased in gastrointestinal tract and lung cancer (Ehlenz et al. 1997).
Aldolase A				✓		1	yes	P04075	1.7E+05	(Morioka 1992)	A housekeeping gene differentially expressed during development and increased in ovarian (Tomonaga et al. 2004) and renal cell cancer (Zhu et al. 1991).
Alkaline phosphatase bone-specific				✓		1	NF	P05186	4.1E+04	(Malati and Yadagiri 2004)	Bone specific alkaline phosphatase may play a role in skeletal mineralization. Bone-specific alkaline phosphatase was significantly increased in prostate cancer patients with bone metastases compared to patients without metastases (Jung et al. 2004).
Alkaline phosphatase, placental type					✓	1	yes	P05187			An oncodevelopmental protein, PLAP was not detected in any of the 22 controls or 12 glioma patients, but high PLAP levels were detected in all 15 germinoma patients, with values ranging from 15 to 3700 pg/ml (Watanabe et al. 2004).
Alpha-1-acid glycoprotein 1, orosomucoid				✓		1	yes	P02763	6.9E+08	(Laboratories 2001)	An acute phase protein showing a 3–4 fold increase during inflammation or tissue damage, levels peak 3–5 days after the initiating event. It is increased in breast cancer (Tesarova et al. 2003).
Alpha-1-antitrypsin				✓		1	yes	P01009	1.4E+09	(Laboratories 2001)	A protease inhibitor and marker of malignant histiocytes (Meyer et al. 1986).
alpha-2-HS-glycoprotein				✓		1	yes	P02765	6.1E+08	(Dickson et al. 1983)	Promotes endocytosis, possesses opsonic properties and plays a role in bone metabolism; it is decreased in leukemia patients (Kwak et al. 2004).
Alpha-2-macroglobulin				✓		1	yes	P01023	1.8E+09	(Laboratories 2001)	A serum plasma proteinase inhibitor with a wide specificity, it is decreased in prostate cancer with metastases (Kanoh et al. 2001).
Alpha-lactalbumin				✓		1	NF	P00709	2.0E+04	(Kolsto Otnaess et al. 1983)	The principle milk protein that functions in the synthesis of lactose, it is increased in some breast cancer patients (Vasil’ev and Avdeev 1985).
Angiogenin ribonuclease RNase A family 5				✓		1	yes	P03950	4.0E+05	(Pavlov and Badet 2001)	An angiogenesis protein increased in pancreatic, stomach, kidney, invasive bladder, colorectal, breast, ovarian, endometrial, uterine, cancer and melanoma (Pavlov and Badet 2001).
Angiopoietin 1				✓		1	yes	Q15389	4.0E+03	(Caine et al. 2003)	Involved in vasculature modeling, it is increased in breast cancer (Caine et al. 2003).
Angiopoietin 2				✓		1	yes	O15123	1.5E+03	(Caine et al. 2003)	It is involved in vasculature modeling in that it is an antagonist of angiopoietin 2. It is increased in breast cancer (Caine et al. 2003).
Antileukoproteinase 1, SLPI				✓		1	yes	P03973	3.2E+04	(Tsukishiro et al. 2005)	An acid-stable proteinase inhibitor with strong affinity for trypsin and chymotrypsin as well as for neutrophil lysosomal elastase and cathepsin G, it is elevated in ovarian cancer patients (Tsukishiro et al. 2005).
Apolipoprotein A1				✓		1	yes	P02647	1.4E+09	(Glowinska et al. 2003b)	Apolipoprotein A-I is the major apoprotein of HDL. ApoA-I also promotes efflux of cholesterol from cell. It is decreased ovarian cancer (Zhang et al. 2004).
Apolipoprotein A-II				✓		1	yes	P02652	2.4E+08	#N/A	Associates with, stabilizes and regulates metabolism of HDL. ApoA-II is overexpressed both in tissues and serum from individuals with prostate cancer. ApoA-II was also overexpressed in the serum of individuals with prostate cancer who have normal prostate-specific antigen (0–4.0 ng/mL) (Malik et al. 2005).
Apolipoprotein C-I				✓		1	yes	P02654	6.1E+07	(Riesen and Sturzenegger 1986)	The smallest of the apolipoproteins, lipid metabolism regulators, the Apo-CI gene is upregulated in gastric cancer (Yasui et al. 2004).
Apolipoprotein C-III				✓		1	yes	P02656	1.2E+08	(Onat et al. 2003)	It delays the catabolism of triglyceride-rich particles and is decreased in myeloid leukemia patients (Kwak et al. 2004).
Bone sialoprotein II				✓		1	yes	P21815	1.5E+05	(Fedarko et al. 2001)	A noncollagenous bone protein increased in prostate, colon, and breast cancer (Fedarko et al. 2001).
Brain-derived neurotrophic factor				✓		1	yes	P23560	2.4E+04	R&D Quantikine kit	It promotes the survival of neuronal populations and is differentially expressed in ovarian cancer (Mor et al. 2005).
Breast cancer metastasis- suppressor 1			✓			1	yes	Q9HCU 9			BRMS1 suppresses metastases. BRMS1 mRNA expression was high in melanocytes, considerably reduced in early melanoma-derived cell lines, and barely detectable in advanced/meta- static cell lines (Shevde et al. 2002).
CA 27.29					✓	1	NF	x			A monoclonal antibody identified cancer antigen most frequently used to follow response to therapy in patients with metastatic breast cancer (Perkins et al. 2003).
CA 72–4					✓	1	NF	x			A monoclonal antibody identified cancer antigen useful in the diagnosis of breast (Skates et al. 2004) and pancreatic cancer (Jiang et al. 2004).
Cathepsin B				✓		1	yes	P07858	2.1E+03	(Kos et al. 1998)	A major cysteine protease involved in antigen degradation, it is overexpressed in tumors of the lung, prostate, colon, breast, stomach and esopha- geal adenocarcinoma (Hughes et al. 1998).
CC chemokine 4, HCC-4				✓		1	yes	O15467	1.1E+04	(Nomiyama et al. 2001)	A chemotactic and myelosuppressive factor, differentially expressed in ovarian cancer (Mor et al. 2005).
CD44 variant V5 soluble				✓		1	yes	P16070	3.3E+04	(Lockhart et al. 1999)	A lymphocyte homing receptor found in the serum of patients with malignant bone tumors (Holzer et al. 2003).
Ceruloplasmin				✓		1	yes	P00450	2.8E+08	(Kim et al. 2002)	A copper binding plasma metalloprotein increased in laryngeal cancer patients (Taysi et al. 2003).
Cervical cancer 1 protooncogene protein p40				✓		1	NF	x	1.5E+07	(Yoon et al. 2004)	A protooncogene expressed in the plasma and tissues of hepatocellular cancer patients (Yoon et al. 2004).
Chemokine (C-C motif) ligand 4 Small inducible cytokine A4 (CCL4), MIP-1-beta				✓		1	yes	P13236	7.0E+01	(Grygorczuk et al. 2003)	CCL4 is a protein that directs the migration of specific subsets of leukocytes. It is elevated in sera from large granular lymphocyte leukemia patients (Kothapalli et al. 2005).
Claudin-3			✓			1	NF	O15551			Claudins are involved in the formation of TJ strands upregulated in ovarian cancer (Lu et al. 2004).
Claudin-4			✓			1	NF	O14493			Claudins are involved in the formation of TJ strands upregulated in ovarian cancer (Hibbs et al. 2004).
Clusterin				✓		1	yes	P10909	1.0E+08	(Hogasen et al. 1993)	Inhibits complement-mediated cytolysis. It is decreased in leukemia patients (Kwak et al. 2004).
Coagulation factor III				✓		1	yes	P13726	1.6E+02	(Mackman 2004)	Coagulation factor III initiates coagulation, it is upregulated in patients with malignancy-associated hypercoagulable state.
Coagulation factor XIII A chain				✓		1	yes	P00488	5.2E+06	(Katona et al. 2000)	The catalytic unit of factor XIII which crosslinks fibrin, is decreased in breast cancer tissues (Jiang et al. 2003).
Coagulation factor XIII B chain				✓		1	NF	P05160	4.8E+06	(Katona et al. 2000)	The protein carrier subunit of factor XIII, it crosslinks fibrin. It is decreased in breast cancer tissues (Jiang et al. 2003).
Collagen I c-terminal telopeptide				✓		1	NF	P02452	2.9E+02	(Malati and Yadagiri 2004)	Collagen is a structural protein, the c-terminal telopeptide is increased in patients with prostate cancer and bone metastasis (Garnero et al. 2000).
Complement component 3				✓		1	yes	x	1.3E+09	(Laboratories 2001)	An effector of innate and adaptive immunity, it is increased in renal carcinoma patients (Holm et al. 1982).
Complement component 4				✓		1	NF	x	2.3E+08	(Sampietro et al. 2004)	An effector of innate and adaptive immunity, it is increased in renal carcinoma patients (Holm et al. 1982).
Complement component 7				✓		1	yes	P10643	5.2E+07	(Laboratories 2001)	An effector of innate and adaptive immunity, its mRNA is decreased in oesophageal, colon and kidney cancers (Oka et al. 2001).
Complement factor H related protein					✓	1	NF	Q03591			Complement factor H related protein is involved in complement regulation. It has a role in cancer surveillance and in the screening of high-risk asymptomatic bladder cancer patients (Quek et al. 2002).
Cyclin-dependent kinase 6			✓			1	yes	Q00534			Cyclin-dependent kinase 6 links growth factor stimulation with the onset of cell cycle progression. Immunohistochemical studies showed reduced levels of cdk6 in breast tumor cells as compared with normal breast tissue in vivo (Lucas et al. 2004).
Cyclooxygenase-2		✓				1	yes	P35354			Cox-2 is induced by inflammation mediators and overexpressed in various cancers (Koga et al. 2004).
Cystatin A				✓		1	yes	P01040	3.2E+03	(Kos et al. 2000)	Inhibitor of the cysteine proteinase cathepsin B, it is increased in squamous cell carcinoma of the head and neck (Strojan et al. 2001).
Cystatin B				✓		1	yes	P04080	1.7E+03	(Kos et al. 2000)	Inhibitor of the cysteine proteinases cathepsin L decreased in squamous cell carcinoma of the head and neck (Strojan et al. 2001).
Cystatin C				✓		1	yes	P01034	3.2E+05	(Strojan et al. 2004b)	The most abundant extracellular inhibitor of cysteine proteases, it is produced in all organs. It is decreased in squamous cell carcinoma of the head and neck (Strojan et al. 2004a) and in serum from ovarian cancer patients however, protein expression in ovarian cancer tissue is increased (Nishikawa et al. 2004).
Cytokeratin 8				✓		1	yes	P05787	5.0E+04	(Fukunaga et al. 2002)	A cytoskeleton protein differentially expressed in pancreatic cancer (Silen et al. 1995).
Diazepam binding inhibitor				✓		1	yes	P07108	1.0E+00	(Avallone et al. 1998)	DBI interacts with GABA receptors downregulating the inhibitory effects of GABA. It participates in the metabolism and genesis of steroids. DBI was found to be increased in the serum from Hepatocellular patients but decreased in their tissue (Venturini et al. 1998).
Endoglin				✓		1	yes	P17813	3.4E+04	(Takahashi et al. 2001)	An angiogenesis factor increased in breast cancer (Li et al. 2000).
Endothelin 1				✓		1	yes	P05305	1.5E+00	(Tsutamoto et al. 1995)	Endothelin is a vasoconstrictor significantly elevated in 80% of primary human colon cancers (Kim et al. 2004).
Epidermal growth factor				✓		1	yes	P01133	1.5E+01	(Oka and Orth 1983)	Epidermal growth factor stimulates the growth of various epidermal and epithelial tissues, it is differentially expressed in ovarian cancer (Mor et al. 2005).
E-selectin				✓		1	yes	P16581	9.2E+02	(Byrne et al. 2000)	An adhesion molecule, sE-selectins increased in metastatic breast cancer especially in patients with liver metastases (Hebbar and Peyrat 2000).
Ferritin H				✓		1	NF	P02794	5.0E+04	Ferritin (Hetet et al. 2003)	An iron storage protein secreted by hepatocellular tumors (Cohen 1988). Several clinical conditions can give rise to increased serum ferritin levels in the absence of high iron stores, including cancer, inflammation, and infection (Hetet et al. 2003). Concentration is for complex protein.
Ferritin, L				✓		1	yes	P02792	5.0E+04	Ferritin (Hetet et al. 2003)	The major intracellular iron storage protein, it is raised in hepatocellular cancer (Cohen 1988), inflammation and infection (Hetet et al. 2003). Concentration is for complex protein.
Fibroblast growth factor 2 (basic)				✓		1	yes	P09038	7.9E+00	(Song et al. 2002)	Fibroblast growth factor is a wide-spectrum mitogenic, angiogenic, and neurotrophic factor elevated in advanced melanoma (Ugurel et al. 2001) and myeloma (Sezer et al. 2001).
Fibronectin 1				✓		1	yes	P02751	4.0E+05	(Hegele et al. 2003)	Thought to have a role in cell adhesion, morphology, surface architecture and contact inhibition. It is increased in renal cell cancer being highest in metastatic disease (Hegele et al. 2004).
Flt-3 ligand				✓		1	yes	x	4.0E+02	(Zwierzina et al. 1999)	Flt-3 ligand promotes long-term expansion, differentiation and proliferation of some hematopoietic cells. Higher pretreatment serum levels of Flt3L in lymphoma are associated with higher stage (> or = II) and higher grade (Retzlaff et al. 2002).
Fms-related tyrosine kinase 1, VEGFR1				✓		1	yes	P17948	3.0E+04	(Caine et al. 2003)	An oncogene that is important for the control of cell proliferation and differentiation, it is reduced in breast cancer (Caine et al. 2003).
Follistatin				✓		1	NF	P19883	6.8E+02	(Hughes and Evans 2003)	An activin antagonist, follistatin inhibits the biosynthesis and secretion of pituitary follicle stimulating hormone. It is differentially expressed in ovarian cancer (Mor et al. 2005).
Fructose- bisphosphate aldolase B				✓		1	NF	P05062	2.0E+04	(Asaka et al. 1988)	A housekeeping gene differentially expressed during development decreased in renal cell cancer (Zhu et al. 1991) and malignant liver tumor patients (Asaka et al. 1988).
Fructose- bisphosphate aldolase C				✓		1	NF	P09972	2.0E+04	(Asaka et al. 1990)	A housekeeping gene differentially expressed during development, it is increased in renal cell cancer (Zhu et al. 1991).
Geminin			✓			1	NF	O75496			Geminin is a potent inhibitor of origin assembly and re-replication in multicellular eukaryotes and is a negative regulator of DNA replication during the cell cycle. Geminin expression is increased in 56% and of colon cancers, 58% of rectal cancers, and 60% of human primary breast cancers (Montanari et al. 2005).
Glucose-6- phosphate isomerase				✓		1	NF	P06744	5.5E+07	(Gomm et al. 1988)	A glycolytic enzyme elevated inovarian cancer (Yeshowardhana and Singh 1985).
Glypican-3, n-terminal				✓		1	yes	P51654	6.5E+02	(Hippo et al. 2004)	Glypican-3 may be involved in the modulation of growth. It is increased in hepatocellular cancer (Hippo et al. 2004).
Growth arrest and DNA-damage- inducible alpha			✓			1	yes	P24522			GADD45A is strongly induced by ultraviolet radiation and alkylating agents and may be an effector of processes that regulate prostate cancer cell survival (Shain 2004).
Immunosuppressive acidic protein				✓		1	NF	x	6.2E+08	(Masuda et al. 1997)	An immunosuppressive molecule and prognostic marker in patients with renal cell carcinoma (Matsumoto et al. 2002).
Insulin-like growth factor 1 (somatomedin C)				✓		1	yes	P01343	2.1E+05	(Stattin et al. 2000)	Insulin-like growth factor 1 plays an important role in growth and development. It is decreased in endometrial cancer (Oh et al. 2004) and Non-Hodgkins’ Lymphoma (Mohnike et al. 1995) but increased in prostate cancer (Stattin et al. 2000).
Insulin-like growth factor 2 (somatomedin A)				✓		1	yes	P01344	3.8E+05	(Oh et al. 2004)	Significantly increased in women with (Oh et al. endometrial cancer 2004) but decreased in children with leukemia, Non-Hodgkins’ Lymphoma (NHL) or solid tumors at the time of diagnosis (Mohnike et al. 1995).
Insulin-like growth factor binding protein 1				✓		1	yes	P24591	1.1E+05	(Chokkalingam et al. 2001)	Insulin-like growth factor binding proteins carry insulin-like growth factor thereby regulating its activity. It is differentially expressed in ovarian cancer (Mor et al. 2005).
Insulin-like growth factor binding protein 2				✓		1	yes	P18065	3.1E+05	(Thierry van Dessel et al. 1996)	Insulin-like growth factor binding proteins carry insulin-like growth factor thereby regulating its activity. IGFBP-2 is elevated in Non-Hodgkins’ Lymphoma (Mohnike et al. 1995).
Insulin-like growth factor binding protein 3				✓		1	yes	P17936	2.5E+06	(Stattin et al. 2000)	Insulin-like growth factor binding proteins carry insulin-like growth factor thereby regulating its activity. It is decreased in endometrial cancer (Oh et al. 2004) and Non-Hodgkins’ Lymphoma (Mohnike et al. 1995) but increased in prostate cancer (Stattin et al. 2000).
Intercellular Adhesion Molecule 1				✓		1	yes	P05362	2.1E+05	(Lei and Johnson 2000)	A lymphocyte adhesion molecule elevated in melanoma (Boyano et al. 2000), hepatocellular cancer (Tsujisaki et al. 1991), breast cancer (Altomonte et al. 1999), and extranodal lymphomas (Lei and Johnson 2000).
Interferon alpha 1				✓		1	yes	P01562	4.5E+01	(Laboratories 2001)	An antiviral cytokine that promotes cell-mediated immunity against intracellular microbes, it is differentially expressed in leukemia (Heyman et al. 1993).
Interleukin 1 alpha				✓		1	yes	P01583	3.0E+00	(Suzuki et al. 1992)	An inflammation and innate immunity modulator, IL-1 alpha is increased in ovarian cancer (Kondera-Anasz et al. 2003).
Interleukin 1 beta				✓		1	yes	P01584	5.0E+00	(Lathers et al. 2003)	IL-1 beta is an inflammation and innate immunity modulator, loss of activity seen in prostate cancer (Ricote et al. 2004).
Interleukin 10				✓		1	yes	P22301	3.5E+00	(Lathers et al. 2003)	IL-10 a suppressive cytokine, is increased in melanoma patients with metastases and poorer prognosis (Boyano et al. 2000).
Interleukin 12A				✓		1	yes	P29459	3.9E+00	(Laboratories 2001)	IL-12 is involved in innate and specific immune responses, levels were lower in malignant glioma (Salmaggi et al. 2003).
Interleukin 16				✓		1	yes	Q14005	1.0E+02	(Alexandrakis et al. 2004)	Interleukin 16 is a chemotactic cytokine increased in multiple myeloma (Alexandrakis et al. 2004).
Interleukin 5				✓		1	yes	P05113	8.0E+00	(Laboratories 2001)	IL-5 is an inflammation marker that links T cell activation with eosinophils which are responsible for clearing of parasites. It is increased in Hodgkin’s disease (Di Biagio et al. 1996).
Interleukin 6 receptor				✓		1	yes	P08887	4.5E+02	(Alexandrakis et al. 2003)	An inflammation marker receptor that regulates the immune response, acute-phase reactions and hematopoiesis, it is significantly elevated in multiple myeloma patients (Alexandrakis et al. 2003). IL6 receptor mRNA was detected in 53% of breast carcinoma tissues and is associated with earlier stages of the disease. In advanced stages, expression of IL-6 and its receptor subunits predicts better prognosis (Karczewska et al. 2000).
Interleukin 6 signal transducer				✓		1	NF	P40189	2.7E+05	(Li et al. 2001)	A signal transducer molecule, increased in breast cancer (Karczewska et al. 2000).
Interleukin 7				✓		1	yes	P13232	1.1E+01	(Xie et al. 2004)	Il-7 stimulates hematopoiesis, it is increased in ovarian cancer (Xie et al. 2004).
Interleukin 8				✓		1	yes	P10145	8.3E+01	(Reinsberg et al. 2000) (Grygorczuk et al. 2003)	Il-8 is a chemotactic factor. Elevated serum concentrations were associated with advanced disease stages and melanoma tumor burden (Ugurel et al. 2001).
Interleukin 9				✓		1	yes	P15248	6.0E+00	(Fischer et al. 2003)	IL-9 supports growth of some immune cells, it is increased in Hodgkin’s lymphoma (Fischer et al. 2003).
Interleukin-1 receptor antagonist protein, IRAP				✓		1	yes	P18510	1.6E+03	(Laboratories 2001)	An acute phase protein that is antagonistic to IL alpha and beta, it is downregulated in oesophageal adenocarcinoma (Hourihan et al. 2003).
Kallikrein 14 (hK14)				✓		1	yes	Q9P0G3	1.6E+02	(Borgono et al. 2003) in male serum female serum negative	Kallikrein 14 is a serine protease increased in 40% of ovarian cancer tissues and elevated in the serum of a proportion of patients with ovarian (65%) and breast (40%) cancers (Borgono et al. 2003).
Kallikrein 2 prostatic				✓		1	NF	P20151	2.2E+01	(Vaisanen et al. 2004)	A serine endopeptidase, kallikrein 2 may predict pathologically organ confined prostate cancer in patients with stage T2 disease but not in stageT1c (Haese et al. 2005).
Kallikrein 5					✓	1	yes	Q9Y337			Kallikrein 5 is a peptidase increased in ovarian cancer tissues (Yousef et al. 2003).
Kallikrein 7					✓	1	NF	P49862			Kallikrein 7 is a peptidase increased in ovarian cancer tissues (Yousef et al. 2003).
Kallikrein 8					✓	1	yes	O60259			Kallikrein 8 is a peptidase increased in ovarian cancer tissues (Yousef et al. 2003).
Keratin 18				✓		1	yes	P05783	4.0E+03	(Ramazan Sekeroglu et al. 2002)	Keratin 18 is one of the first intermediate filament proteins expressed in the embryo. A monoclonal antibody to epithelium-specific keratin 18 stained the majority of inner cells in benign breast lesions but comparatively fewer such cells in carcinoma in situ and invasive carcinoma (Rudland et al. 1993).
Keratin, type I cytoskeletal 19, cytokeratin 19				✓		1	yes	P08727	2.4E+03	(Hasholzner et al. 1994)	A cytoskeleton protein increased in bladder (Morita et al. 1997) and breast cancer (Grunewald et al. 2000).
Kit ligand				✓		1	yes	P21583	3.3E+05	(Bono et al. 2004)	A hematopoietic growth factor, decreased in patients with gastrointestinal stromal tumors (Bono et al. 2004).
Lactotransferrin				✓		1	yes	P02788	2.7E+05	(Vasil’ev and Avdeev 1985)	An iron-binding protein that modulates iron metabolism, hematopoiesis, and immunologic reactions. It is increased in gastrointestinal, lung and breast cancer patients (Vasil’ev and Avdeev 1985).
Leptin				✓		1	yes	P41159	4.8E+03	(Doehner et al. 2001)	Leptin plays a critical role in the regulation of body weight and is decreased in gastrointestinal carcinomas (Dulger et al. 2004).
L-selectin				✓		1	yes	P14151	6.0E+05	(Atalar et al. 2001)	An adhesion molecule elevated in non-Hodgkin’s lymphoma and Hodgkin’s disease (Haznedaroglu et al. 2000).
Luteinizing hormone-releasing hormone receptor					✓	1	NF	x			A growth inhibiting tyrosine phosphatase found in 29 of 37 (78.4%) ovarian cancers and in 6 of 11 (54.5%) non-malignant human ovaries (Srkalovic et al. 1998).
Mac-2 Binding Protein 90K				✓		1	NF	Q08380	9.1E+06	(Iacovazzi et al. 2001)	Promotes integrin-mediated cell adhesion, it is increased in breast (Iacobelli et al. 1994) and hepatocellular cancer (Iacovazzi et al. 2001).
Mammaglobin B			✓			1	NF	O75556			Mammaglobin B may bind androgens and other steroids, it shows high sequence similarity to mammaglobin. Frequently upregulated in lung tumors (Sjodin et al. 2003).
Mammary Serum Antigen						0	NF	x	4.0E+05	(Smart et al. 1990)	A serum glycoprotein on breast cancer cells detectable in serum. It may be an early prognostic marker in breast cancer (Smart et al. 1990).
Mast/stem cell growth factor receptor				✓		1	NF	P10721	3.3E+05	(Bono et al. 2004)	A proto-oncogene tyrosine-protein kinase expressed in acute promyelocytic leukemia (Rizzatti et al. 2002) and in the serum of gastrointestinal stromal tumor patients (Bono et al. 2004).
Matrix metalloproteinase 2				✓		1	yes	P08253	1.0E+05	(Sasaki et al. 2002)	A metalloproteinase that specifically cleaves type IV collagen, the major structural component of basement membranes. The metastatic potential of tumor cells has been found to correlate with the activity of this enzyme. It is markedly elevated in colorectal cancer patients (Doubrovina et al. 2003).
Matrix metalloproteinase 9				✓		1	yes	P14780	1.9E+05	(Riedel et al. 2000)	MMPs are capable of disintegrating the basement membrane, which is a main characteristic of tumor invasion. MMP9 was significantly increased in patients with squamous cell cancer of the head and neck over controls. It was not changed in inflammatory diseases (Kuropkat et al. 2002).
Melanoma-inhibiting activity				✓		1	NF	Q16674	8.8E+03	(ElGuba et al. 2002)	A protein secreted by malignant melanoma cells that elicits growth inhibition of melanoma cells in vitro. Elevated levels predict a poor prognosis (ElGuba et al. 2002).
Membrane cofactor protein, CD46 antigen				✓		1	NF	P15529	3.5E+04	(Seya et al. 1995)	A membrane protein that protects host cells from complement damage. Normal human sera contained 10–60 ng/ml of soluble membrane cofactor protein whereas sera of > 50% of cancer patients contained > 60 ng/ml (Seya et al. 1995).
Mesothelin					✓	1	NF	Q13421			A differentiation antigen, overexpressed in several human tumors (Hassan et al. 2004).
Midkine				✓		1	NF	P21741	1.5E+02	(Ikematsu et al. 2000)	Midkine has heparin binding activity, and growth promoting activity, it is increased in breast cancer patients (Ikematsu et al. 2000).
MK-1 protein, Ep-CAM				✓	✓	1	yes	x	2.0E+03	(Abe et al. 2002)	A membrane glycoprotein that is overexpressed on the majority of tumor cells of epithelial origin, it is increased in the serum from patients with malignant tumors of various tissue origins (Abe et al. 2002).
Myoblast determination protein 1			✓			1	yes	P15172			A myogenic transcriptional regulatory protein expressed early in skeletal muscle differentiation, it is considered a sensitive and specific marker for Rhabdomyosarcoma and is more specific than desmin and muscle-specific actin and more sensitive than myoglobin (Cessna et al. 2001).
Nerve growth factor beta				✓		1	yes	P01138	7.0E+02	(Reynolds et al. 2003)	Nerve growth factor is important for the development and maintenance of the sympathetic and sensory nervous systems. Immunostaining for nerve growth factor-beta in esophageal and breast carcinomas demonstrated its immunoreactivity in stromal fibroblasts and some TrkA-expressing tumor cells (Koizumi et al. 1998).
Netrin-1			✓			1	NF	O95631			Signals axon growth and guidance. A reduction of NTN1 expression was observed in prostate tumors (Latil et al. 2003).
Neuroendocrine secretory protein-55			✓			1	NF	x			A peptidergic marker for a large constitutively secreting vesicle pool found in the central and peripheral nervous system, NESP-55 reactivity is restricted to endocrine tumors of the pancreas and the adrenal medulla (Srivastava et al. 2004).
Neutrophil defensin 1				✓		1	yes	P59665	4.2E+04	all defensins measured together (Panyutich et al. 1993)	An antimicrobial protein secreted by neutrophils increased in colon cancer patients (Albrethsen et al. 2005). Concentration given is for all three defensins together 1,2 & 3.
Neutrophil defensin 3				✓		1	yes	P59666	4.2E+04	all defensins measured together (Panyutich et al. 1993)	An antimicrobial protein secreted by neutrophils increased in colon cancer patients (Albrethsen et al. 2005). Concentration given is for all three defensins together 1,2 & 3.
Nm23-H1				✓		1	yes	P15531	6.1E+03	(Okabe-Kado 2002)	The metastasis-suppressor protein, nucleoside diphosphate kinase A is increased in the serum of patients with hematological neoplasms (Okabe-Kado 2002).
OVX1					✓	1	NF	x			An ovarian cancer antigen antibody, OVX1 reacted to a majority of ovarian cancer tissues (17 of 20) and did not bind to normal ovarian tissues. (Xu et al. 1991).
OX40				✓		1	yes	P43489	7.5E+02	(Taylor and Schwarz 2001).	Ox40 helps maintain T cell responses. Solubl OX40 is detectable in serum of subpopulations of healthy donors and patients with autoimmune disease and cancer. Chronic lymphocytic leukemia has been identified as a disease with high frequency of sOX40-positive sera and with the highest mean sOX40 serum concentration (Taylor and Schwarz 2001).
p65 oncofetal protein				✓		1	NF	x	3.7E+04	(Mirowski et al. 1994)	A novel member of the superfamily of genes that encode nuclear receptors for various hydro-phobic ligands such as steroids, vitamin D, retinoic acid, and thyroid hormones, it is increased in 90% of breast cancer patients (Mirowski et al. 1994).
Pancreatic secretory trypsin inhibitor, TATI				✓		1	NF	P00995	2.1E+04	(Medl et al. 1995)	It is secreted from pancreatic acinar cells into pancreatic juice. Its physiologic role has been thought to be the prevention of trypsin-catalyzed premature activation of zymogens within the pancreas and the pancreatic duct. Since it is also found in serum and in various normal and malignant tissues, it may have other roles as well. It is elevated in ovarian cancer (Medl et al. 1995).
Parathyroid hormone-related protein					✓	1		P12272			A critical regulator of cellular and organ growth, development, migration, differentiation and survival and of epithelial calcium ion transport; parathyroid hormone-related protein is found in the serum of bone metastases (Iguchi et al. 2004), lung cancer (Nishigaki et al. 1999) patients and a multiple myeloma patient (Kitazawa et al. 2002).
Pcaf, P300/CBP-associated factor			✓			1	yes	Q92831			Pcaf plays a direct role in transcriptional regulation. The genes for p300, CBP, MOZ and MORF are rearranged in recurrent leukemia-associated chromosomal abnormalities (Yang 2004).
Pepsinogen-1				✓		1	NF	x	4.4E+04	(Gao and Zhang 2004)	The precursor of pepsin, one of the main proteolytic enzymes secreted by the gastric mucosa, it is decreased in gastric cancer patients (Konturek et al. 2003).
Placental specific tissue protein 12				✓		1	NF	x	5.4E+01	(Briese et al. 1986)	A soluble tissue antigen of the placenta, it is increased in lung cancer (Briese et al. 1986).
Plasma retinol-binding protein				✓		1	NF	P02753	3.2E+07	(Laboratories 2001)	The specific carrier for vitamin A in the blood, it is decreased in leukemia patients (Kwak et al. 2004).
Plasminogen (Contains Angiostatin)				✓		1	NF	P00747	1.1E+08	(Laboratories 2001)	The precursor to angiostatin, a potent angiogenesis inhibitor, it is increased in patients with malignant neoplasm of stomach, colon, lung, bladder, breast. renal pelvis, and prostate but decreased in patients with malignant neoplasm of biliary tree, pancrease, cervix uteri, kidney except pelvis, and thyroid (Chang Kyou et al. 2004).
Platelet endothelial cell adhesion molecule, PECAM-1				✓		1	yes	P16284	6.6E+03	(Zeisler et al. 2001)	Involved in transendothelial migration of leukocytes, angiogenesis, and integrin activation; it is underexpressed in adenocarinomas of the lung (McDoniels-Silvers et al. 2002) but decreased in patients with recurrent basal cell carcinoma (Yerebakan et al. 2003).
Platelet factor 4				✓		1	NF	P02776	9.7E+03	(Leitzel et al. 1991)	Promotes coagulation and plays a role in inflammation and wound repair, it is elevated in some cancer patients (Leitzel et al. 1991).
Platelet-derived growth factor beta polypeptide				✓		1	yes	P01127	3.2E+02	(Leitzel et al. 1991)	A potent mitogen for cells of mesenchymal origin, all gliomas expressed PDGF-B mRNA at higher levels than found in peritumoral and normal nervous tissues (Mauro et al. 1991).
Platelet-derived growth factor receptor alpha polypeptide			✓			1	NF	P16234			Mediates various growth factors. By Western blot analysis, PDGFR protein expression was detected in 10 of 11 basal cell carcinomas, whereas it was undetectable in the control epidermis (Xie et al. 2001).
Pregnancy zone protein				✓		1	NF	P20742	8.4E+06	(Petersen et al. 1990)	A prominent constituent of late-pregnancy sera, it is increased in gynaecological tumors (Teng et al. 1994).
Pregnancy- associated plasma protein-A				✓		1	NF	Q13219	1.0E+03	(Qin et al. 1997)	PAPP-A can bind a variety of cytokines and specifically cleave a binding protein for insulin-like growth factors, thereby serving as a modulator of cytokine activity. It is increased in breast cancer (Kuhajda and Eggleston 1985).
Prostate secretory protein PSP94				✓		1	yes	P08118	7.1E+05	(Reeves et al. 2005)	Inhibits follicle-stimulating-hormone secretion, PSP94 serum measurements, especially of bound and free forms, have potential clinical utility in prostate cancer management (Reeves et al. 2005).
P-selectin				✓		1	yes	P16109	1.9E+05	(Atalar et al. 2001)	An adhesion molecule that mediates the interaction of activated endothelial cells or platelets with leukocytes, it is elevated in Hodgkin’s and non-Hodgkin’s lymphoma (Haznedaroglu et al. 2000).
PSP94 binding protein				✓		1	yes	x	7.1E+05	(Reeves et al. 2005)	It may be involved in hormonal control. It is lower in the serum of prostate cancer patients (Reeves et al. 2005).
Pyruvate kinase, isozymes M1/M2				✓		1	NF	P14618	1.5E+07	(Luftner et al. 2000)	May play a role in metabolism, an isoenzyme of pyruvate kinase it is overexpressed by some tumor cells including pancreatic tumors (Ventrucci et al. 2004).
Riboflavin carrier protein				✓		1	NF	x	7.0E+02	(Rao et al. 1999)	Riboflavin carrier proteins transports vitamin B2 across placental membranes, a process critical for maintenance of pregnancy. It is 3 to 4-fold higher in breast cancer patients. In addition, there seems to be a good correlation between rising RCP levels and disease progression (Karande et al. 2001).
S100 beta chain				✓		1	NF	P04271	9.0E+01	(Tas et al. 2004)	S100 binds zinc and calcium. A statistically significant shorter survival was found in patients with high levels (Vos et al. 2004). Concentration is for the complex protein.
Secreted phosphoprotein 1, osteopontin				✓		1	yes	P10451	4.4E+05	(Fedarko et al. 2001)	An extracellular matrix protein of pleiotropic properties including inflammation modulator, it is increased in prostate, colon, breast and lung cancer (Fedarko et al. 2001).
Serine (or cysteine) proteinase inhibitor clade B, maspin				✓		1	NF	P05154	5.3E+06	(Laurell et al. 1992)	A tumor suppressor decreased in stomach cancer.
Serine (or cysteine) proteinase inhibitor clade E, PAI-1				✓		1	NF	P05121	8.0E+03	(Koong et al. 2000)	It inhibits tissue plasminogen activator, urokinase, and protein C. PAI-1 levels were measured in the serum of a small group of head and neck cancer patients and were found to correlate with the degree of tumor hypoxia found in these patients (Koong et al. 2000).
Serum amyloid alpha-1				✓		1	yes	P02735	3.6E+07	(Fyfe et al. 1997)	The proteolytic cleavage product of an acute phase reactant, it is differentially expressed in renal cancer (Tolson et al. 2004).
Serum paraoxonase/ arylesterase 1				✓		1	NF	P27169	5.9E+07	(Kujiraoka et al. 2000)	Hydrolyzes the toxic metabolites of a variety of organophosphorus insecticides. It is decreased in gastric (Akcay et al. 2003b) and pancreatic (Akcay et al. 2003a) cancer patients.
Small inducible cytokine A14 CCL14				✓		1	yes	Q16627	8.5E+03	(Struyf et al. 2003)	CCL14 enhances proliferation of CD34 positive stem cells, it is differentially expressed in lobular versus ductal tumors (Korkola et al. 2003).
Small inducible cytokine A18(CCL18), MIP-4				✓		1	NF	P55774	3.1E+04	(Struyf et al. 2003)	An immune modulator that governs antigen presenting dendritic cells and immature T cells.
Small inducible cytokine A2(CCL2)				✓		1	yes	P13500	1.8E+02	(Lebrecht et al. 2004)	CCL2 is a monocyte, chemotactic and activating factor increased in some breast cancer (Lebrecht et al. 2004).
Small inducible cytokine A3(CCL3), Macrophage inflammatory protein 1-alpha				✓		1	yes	P10147	3.7E+01	(Grygorczuk et al. 2003)	MIP-1 alpha is an immunoregulatory and inflammatory molecule increased in myeloma (Terpos et al. 2003).
Small inducible cytokine B5(CXCL5)				✓		1	yes	P42830	4.0E+02	(Doubrovina et al. 2003)	CXCL5 is involved in neutrophil activation. Its gene expression is suppressed in malignant nasopharyngeal epithelial cells (Fung et al. 2000).
Squamous cell carcinoma antigen 1				✓		1	NF	P29508	4.2E+03	(Cataltepe et al. 2000)	A member of the ovalbumin family of serine proteinase inhibitors, it serves as a serologic marker for advanced squamous cell carcinomas of the uterine cervix, lung, esophagus, head and neck and vulva. Recent molecular studies show that SCCA is transcribed by two nearly identical genes (SCCA1 and SCCA2) that encode for members of the high molecular weight serine proteinase inhibitor (serpin) family (Cataltepe et al. 2000).
Squamous cell carcinoma antigen 2				✓		1	NF	P48594	2.0E+03	(Senekjian et al. 1987)	SCCA2 may act as a protease inhibitor to modulate the host immune response against tumor cells. It is significantly elevated in cervical cancer (Barnes et al. 2000).
Survivin				✓		1	yes	O15392	1.2E+02	(Bokarewa et al. 2005)	Survivin is an apoptosis inhibitor upregulated in adult T cell leukemia and acute leukemia but downregulated in chronic lymphocytic leukemia (Sugahara et al. 2004).
Syndecan-1				✓		1	NF	P18827	4.0E+04	(Kyrtsonis et al. 2004)	A cell surface proteoglycan, it is an integral membrane protein acting as a receptor for the extracellular matrix. It is expressed in most multiple myeloma patients (Kyrtsonis et al. 2004).
synuclein-gamma			✓			1	NF	O7607			Synuclein-gamma plays a role in neurofilament network integrity. It is found in sera from 21 of 56 pancreatic patients (Li et al. 2004).
TEK tyrosine kinase endothelial, Tie-2				✓		1	yes	Q02763	8.5E+03	(Chung et al. 2003)	Tie-2 is involved in angiogenesis, vasculogenesis and hematopoiesis. Increased in breast and prostate cancer (Caine et al. 2003).
Tenascin				✓		1	yes	P24821	1.0E+06	(Schienk et al. 1995)	An extracellular matrix protein with a spatially and temporally restricted tissue distribution, it is elevated in cancer patients especially patients with high C-reactive protein levels (Schenk et al. 1995).
Tetranectin				✓		1	NF	P05452	8.2E+06	(Hogdall et al. 2002)	A plasma protein that has a specific binding affinity for sulfated polysaccharides and the kringle 4 of plasminogen, it is an independent prognostic factor in ovarian cancer (Begum et al. 2004).
TGF-beta receptor type III			✓			1	NF	Q03167			A TGF-beta binding protein, it may retain TGF-beta for the signaling receptors. It is differentially expressed in ovarian cancer (Mor et al. 2005).
Thioredoxin reductase 1, cytoplasmic				✓		1	NF	Q16881	1.8E+04	(Soderberg et al. 2000)	A redox-active protein that participates in multiple cellular events, including growth promotion, apoptosis, and cytoprotection; it is over expressed leukemia dna melanoma (Soderberg et al. 2000).
Thrombopoietin				✓		1	yes	P40225	7.8E+04	(Hellstrom-Lindberg et al. 1999)	Thrombopoietin may indirectly enhance erythropoiesis. It is increased in acute myeloblastic leukemia and myelodysplastic syndrome (Hsu et al. 2002).
Thrombospondin 1				✓		1	NF	P07996	2.1E+05	(Hayden et al. 2000)	Thrombospondin I is a multimodular secreted protein that associates with the extracellular matrix and possesses a variety of biologic functions, including a potent angiogenic activity. Staining for thrombospondin is positive in hepatocellular carcinoma and a prognostic marker of poor survival (Poon et al. 2004).
Thymidine kinase, cytosolic				✓		1	yes	P04183	1.0E+01	(Di Raimondo et al. 2001)	Thymidine kinase is a DNA replication enzyme that can provide prognostic information on progression-free survival in leukemia patients (Hallek et al. 1996).
Tissue inhibitor of metalloproteinase 1				✓		1	yes	P01033	9.5E+04	(Noji et al. 2001)	A modulator of interstitial collagenase as well as a number of connective tissue metalloendoproteases, TIMPs can form complexes with extracellular matrix metalloproteinases (such as collagenases) and irreversibly inactivate them. The plasma concentration of TIMP-1 in colorectal carcinoma correlates with both invasion and metastasis (Yukawa et al. 2001).
Tissue inhibitor of metalloproteinase 2				✓		1	yes	P16035	3.4E+04	(Noji et al. 2004)	TIMPs can form complexes with extracellular matrix metalloproteinases (such as collagenases) and irreversibly inactivate them. TIMP-2 is reduced in prostate cancer (Lichtinghagen et al. 2003).
Tissue-type plasminogen activator, tPA				✓		1	yes	P00750	7.3E+03	(Glowinska et al. 2003a)	Tissue plasminogen activator is a serine protease that activates the proenzyme plasminogen to plasmin, which in turn is responsible for fibrinolytic activity. tPA is decreased in gastric neoplastic tissues (Sanz et al. 2002).
Transferrin receptor (p90 CD71)				✓		1	NF	P02786	5.2E+06	(Looker et al. 1999)	TFRC is a ubiquitously distributed antigen on the cell surface of actively growing human cell. It is upregulated on neuroendocrine carcinomas of the pancrease(Ryschich et al. 2004) and a sensitive serum measurement of erythropoiesis and iron deficiency (Shih et al. 1990).
Transforming growth factor alpha				✓		1	yes	P01135	1.5E+01	(Chien et al. 1997)	TGF-alpha, a potent mitogenic polypeptide, is present in most gallbladder carcinoma tissue (Lee 1998) and the plasma of ovarian cancer patients (Chien et al. 1997).
Transforming growth factor beta 1				✓		1	yes	P01137	1.4E+04	(Shirai et al. 1994) (Shariat et al. 2001) (Eder et al. 1996)	A transforming growth factor, regulated at the protein level with both inhibitory and stimulatory activities. TGF-beta 1 levels are increased in patients with prostate lymph node and bone metastases (Shariat et al. 2001), invasive bladder cancer (Eder et al. 1996) and cervical cancer (Dickson et al. 2000).
transthyretin				✓		1	NF	P02766	3.0E+08	(Vatassery et al. 1991)	A thyroid hormone binding protein decreased in ovarian cancer patients (Zhang et al. 2004).
Tropomyosin 1 alpha chain (Alpha-tropomyosin)				✓		1	NF	P09493	2.0E+03	(Cummins et al. 1981)	Tropomyosins are ubiquitous proteins of 35 to 45 kD associated with the actin filaments of myofibrils and stress fibers. It is decreased in pancreatic cancer (Alaiya et al. 2000).
Tumor necrosis factor (ligand) superfamily member 5, CD154				✓		1	NF	P29965	1.3E+02	(Roselli et al. 2004)	CD154 is a B cell stimulator increased in lung cancer (Roselli et al. 2004).
Tumor necrosis factor (ligand) superfamily member 6, Fas ligand				✓		1	yes	P48023	2.5E+04	(Sakai et al. 1999)	An apoptosis mediator increased in leukemia, lymphoma (Tanaka et al. 1996), and gastric carcinoma patients (Tsutsumi et al. 2000).
Tumor necrosis factor ligand superfamily member 13B, TALL-1				✓		1	yes	Q9Y275	2.5E+03	(Moreaux et al. 2004)	It can induce activation, proliferation, differentiation, or death in B cells. It is elevated in the serum of patients with systemic autoimmune diseases and in patients with B-lymphoid malignancies (Mackay and Tangye 2004).
Tumor necrosis factor receptor superfamily member 11B, osteoprotegerin				✓		1	yes	O00300	3.5E+01	(Kyrtsonis et al. 2004)	A secreted glycoprotein that regulates bone resorption, osteoprotegerin is increased in patients with bone metastases (Jung et al. 2004) and multiple myeloma (Kyrtsonis et al. 2004).
Tumor necrosis factor receptor superfamily member 1A p60 TNF-RI p55 CD120a, TNFR1				✓		1	yes	P19438	9.1E+02	(Ammirato et al. 2001)	An immune modulator receptor elevated in patients with malignant astrocytomas of the brain (Ammirato et al. 2001).
Tumor necrosis factor receptor superfamily member 1B, TNFR2				✓		1	yes	P20333	4.0E+03	(Tziakas et al. 2004)	The main TNF receptor found on circulating T cells, it is the major mediator of autoregulatory apoptosis in CD8+ cells. TNFR2 may act with TNFR1 to kill nonlymphoid cells. It is elevated in patients with malignant astrocytomas of the brain (Ammirato et al. 2001).
Urokinase plasminogen activator surface receptor, U-PAR				✓		1	yes	Q03405	3.0E+03	(Riisbro et al. 2002)	The urokinase-type plasminogen activator receptor is a key molecule in the regulation of cell-surface plasminogen activation. It is increased in colorectal cancer and associated with poor prognosis in patients with metatatic breast cancer (Begum et al. 2004).
Vascular cell adhesion molecule 1				✓		1	yes	P19320	4.8E+05	(Byrne et al. 2000)	VCAM-1 mediates the adhesion of monocytes and lymphocytes to cytokine-activated endothelium. It is correlated with microvessel density in early breast cancer tumors and increased in women with lymph node-positive and high-grade breast tumors (Byrne and Bundred 2000).
Vascular endothelial growth factor receptor 2				✓		1	yes	P35968	1.5E+04	(Robak et al. 2003)	The VEGF-flk-1 system takes part in tumor angiogenesis, proliferation, and apoptosis in colon liver metastases (Cheng et al. 2004).
Vasoactive intestinal peptide					✓	1	NF	P01282			VIP causes vasodilation, lowers arterial blood pressure, stimulates myocardial contractility, increases glycogenolysis and relaxes the smooth muscle of trachea, stomach and gall bladder. It is increased two fold in adenocarcinoma patients (Collado et al. 2005).
VEGF(165)b				✓		1	NF	x	4.2E+01	(Woolard et al. 2004)	Possesses anti-angiogenic action. This isoform was present in 17 of 18 normal kidney samples but only 4 of 18 cases from matched malignant tissue (Bates et al. 2002).
Vitamin K dependent protein C				✓		1	NF	P04070	3.7E+06	(Kalafatis et al. 1997)	A vitamin K-dependent serine protease that regulates blood coagulation, it is differentially expressed in ovarian cancer (Mor et al. 2005).
Vitronectin				✓		1	NF	P04004	3.4E+05	(Hogasen et al. 1993)	Vitronectin promotes attachment and spreading of animal cells in vitro, it inhibits cytolysis by the complement C5b-9 complex, and modulates antithrombin III-thrombin action in blood coagulation. It is upregulated in colorectal carcinoma (Tomasini-Johansson et al. 1994).
X box binding protein-1			✓			1	NF	P17861			A transcription factor essential for hepatocyte growth, the differentiation of plasma cells, immunoglobulin secretion, and the unfolded protein response. It is increased in identical twins with multiple myeloma(Munshi et al. 2004) hXBP-1 mRNA expression was increased in primary breast cancers but hardly detectable in non-cancerous breast tissue (Fujimoto et al. 2003).

## Abbreviations

MS: mass spectrometry
GO: Genome Ontology
